# Fe(III) (oxyhydr)oxide reduction by the thermophilic iron-reducing bacterium *Desulfovulcanus ferrireducens*

**DOI:** 10.3389/fmicb.2023.1272245

**Published:** 2023-10-20

**Authors:** Elizabeth C. Sklute, Deborah A. Leopo, Kaylee A. Neat, Kenneth J. T. Livi, M. Darby Dyar, James F. Holden

**Affiliations:** ^1^Planetary Science Institute, Tucson, AZ, United States; ^2^Los Alamos National Laboratory, Los Alamos, NM, United States; ^3^Department of Microbiology, University of Massachusetts, Amherst, MA, United States; ^4^Department of Astronomy, Mount Holyoke College, South Hadley, MA, United States; ^5^Department of Materials Science and Engineering, Johns Hopkins University, Baltimore, MD, United States

**Keywords:** thermophile, iron reduction, ferrihydrite, akaganeite, lepidocrocite, magnetite, reflectance spectroscopy, Mössbauer spectroscopy

## Abstract

Some thermophilic bacteria from deep-sea hydrothermal vents grow by dissimilatory iron reduction, but our understanding of their biogenic mineral transformations is nascent. Mineral transformations catalyzed by the thermophilic iron-reducing bacterium *Desulfovulcanus ferrireducens* during growth at 55°C were examined using synthetic nanophase ferrihydrite, akaganeite, and lepidocrocite separately as terminal electron acceptors. Spectral analyses using visible-near infrared (VNIR), Fourier-transform infrared attenuated total reflectance (FTIR-ATR), and Mössbauer spectroscopies were complemented with x-ray diffraction (XRD) and transmission electron microscopy (TEM) using selected area electron diffraction (SAED) and energy dispersive X-ray (EDX) analyses. The most extensive biogenic mineral transformation occurred with ferrihydrite, which produced a magnetic, visibly dark mineral with spectral features matching cation-deficient magnetite. *Desulfovulcanus ferrireducens* also grew on akaganeite and lepidocrocite and produced non-magnetic, visibly dark minerals that were poorly soluble in the oxalate solution. Bioreduced mineral products from akaganeite and lepidocrocite reduction were almost entirely absorbed in the VNIR spectroscopy in contrast to both parent minerals and the abiotic controls. However, FTIR-ATR and Mössbauer spectra and XRD analyses of both biogenic minerals were almost identical to the parent and control minerals. The TEM of these biogenic minerals showed the presence of poorly crystalline iron nanospheres (50–200 nm in diameter) of unknown mineralogy that were likely coating the larger parent minerals and were absent from the controls. The study demonstrated that thermophilic bacteria transform different types of Fe(III) (oxyhydr)oxide minerals for growth with varying mineral products. These mineral products are likely formed through dissolution-reprecipitation reactions but are not easily predictable through chemical equilibrium reactions alone.

## Introduction

Fe(III) oxide mineral phases are found in the interior of deep-sea hydrothermal metal sulfide deposits in the seawater-hydrothermal fluid mixing zone, where O_2_-containing seawater oxidizes iron sulfide minerals (Kristall et al., 2006; Lin et al., 2014, 2016b). These Fe(III) oxide minerals serve as terminal electron acceptors for thermophilic and hyperthermophilic iron-reducing bacteria and archaea that use H_2_ and CO_2_ from the hydrothermal fluid or organic compounds as their carbon and energy sources (Slobodkin et al., 2001; Tor and Lovley, 2001; Kashefi et al., 2003; Miroshnichenko et al., 2003; Slobodkina et al., 2009a,b, 2012; Ver Eecke et al., 2009; Lin et al., 2014; Kashyap et al., 2022). However, our understanding of the types of iron oxides that support the growth of high-temperature microbes and the biogenic minerals produced is limited. This information would help determine the *in situ* locations of these organisms, their biogeochemical effect, and the biotic-abiotic mechanisms of mineral transformation and provide a biosignature.

Ferrihydrite (Fe(OH)_3_) is the least crystalline, and thus the most reactive, Fe(III) (oxyhydr)oxide in a gradient that includes (from least to most crystalline) lepidocrocite (γ-FeOOH), akaganeite (β-FeOOH), goethite (α-FeOOH), and hematite (α-Fe_2_O_3_) (Cornell and Schwertmann, 2003; Kappler et al., 2021). Nanophase Fe(III) (oxyhydr)oxide minerals (< 100 nm in at least one direction) are also more reactive than their microparticulate counterparts due to an increase in reactive surface area (Braunschweig et al., 2013; Kappler et al., 2021). For the iron-reducing bacterium *Geobacter sulfurreducens*, Cutting et al. (2009) found a correlation between the crystallinity of a given synthetic nanophase Fe(III) (oxyhydr)oxide and the rate of Fe(III) reduction. In that study, ferrihydrite was rapidly transformed to magnetite (Fe_3_O_4_), and lepidocrocite and akaganeite were reduced at slower rates to a mixture of goethite and magnetite, with an increasing proportion of magnetite produced with time. The most crystalline oxides, goethites, and hematites were relatively recalcitrant toward the metabolic activity of *G. sulfurreducens*, although a small proportion of the latter two phases was bioavailable. Microbial ferrihydrite reduction to primary magnetite is attributed to the conversion of mineral surface layers to more crystalline phases during Fe(II)-induced remineralization processes. However, this secondary mineralization does not occur following the reduction of goethite and hematite (Hansel et al., 2003, 2004).

The reactivity and biogenic reduction of Fe(III) (oxyhydr)oxides at high temperatures by thermophiles may differ from mesophiles due to biological and chemical differences. The thermophilic bacterium *Desulfovulcanus ferrireducens* and the hyperthermophilic archaeon *Pyrodictium delaneyi* were both isolated from deep-sea hydrothermal vents and grown on various Fe(III) (oxyhydr)oxide minerals at 55°C and 90°C, respectively (Kashyap et al., 2018, 2022). They each produced ~20 mM acid-soluble Fe^2+^ when grown on ferrihydrite and 2–3 mM acid-soluble Fe^2+^ when grown separately on lepidocrocite and akaganeite but grew poorly on hematite and goethite. Geochemical modeling prediction that ferrihydrite reduced to 20 mM Fe^2+^ in the growth medium used for *D. ferrireducens* and *P. delaneyi* would yield mostly magnetite at 55 and 90°C if hematite and goethite formation were suppressed (Supplementary Table 1). In contrast, FeOOH reduced to 5 mM Fe^2+^ would be transformed mostly to magnetite at 90°C but mostly to siderite (FeCO_3_) at 55°C (Supplementary Table 1), suggesting a strong temperature dependence on the mineral product of more crystalline iron oxides. Magnetite was the mineral product when ferrihydrite was reduced at 90°C by *P. delaneyi* based on selected-area electron diffraction (SAED) and visible-near infrared (VNIR), Fourier-transform infrared attenuated total reflectance (FTIR-ATR), Raman, and Mössbauer spectroscopy analyses (Lin et al., 2014; Kashyap et al., 2018, 2023). When *P. delaneyi* was grown at 90°C, some magnetite and a vivianite [Fe_3_(PO_4_)_2_·8H_2_O]-like ferrous phosphate mineral formed when akaganeite was reduced; however, siderite, not magnetite, was formed when lepidocrocite was reduced at 90°C based on spectroscopy analyses (Kashyap et al., 2023).

This study aims to identify the biogenic mineral products formed when *D. ferrireducens* is grown at 55°C separately on ferrihydrite, akaganeite, and lepidocrocite. Specifically, the objective of the study is to determine if only magnetite, especially a bioreduced or cation-deficient form, is formed as predicted when *D. ferrireducens* is grown on ferrihydrite. Another goal is to determine if only siderite is formed when *D. ferrireducens* is grown on akaganeite and lepidocrocite at 55°C. The study will help determine the mechanism used for iron reduction by *D. ferrireducens* and the potential influence of temperature and cell type (i.e., thermophilic bacterium vs. hyperthermophilic archaeon) on iron reduction. Mineral spectroscopy, X-ray diffraction (XRD), and electron microscopy were coupled to provide complementary analyses of the minerals present in bioreduced samples following growth and in uninoculated controls.

## Materials and methods

### Microorganism and incubation conditions

*Desulfovulcanus ferrireducens* strain Ax17^T^ (DSM 111878) was used for this study and was obtained from the Deutsche Sammlung von Mikroorganismen und Zellkulturen (Braunschweig, Germany). The medium used for growth was modified DSM 981 medium (Kashefi et al., 2002). The base medium was composed of the following (per liter): 19.0 g of NaCl, 9.0 g of MgCl_2_ · 6H_2_O, 0.3 g of CaCl_2_ · 2H_2_O, 0.5 g of KCl, 0.14 g of KH_2_PO_4_, 0.05 g of NaBr, 0.02 g of SrCl_2_ · 6H_2_O, 0.15 g of MgSO_4_ · 7H_2_O, 0.1 g of (NH_4_)_2_SO_4_, 1 g of NaHCO_3_, 10 ml of DSM141 Trace Mineral solution, and 10 ml of DSM141 Vitamin solution. Synthetic nanophase ferrihydrite (2-line), akaganeite, and lepidocrocite were synthesized as previously described (Sklute et al., 2018). Then, 100 mmol L^−1^ of each mineral was used separately as terminal electron acceptors. The pH of each medium was adjusted to 6.80 ± 0.05 using 1 N NaOH. All incubations were carried out in 160 ml serum bottles containing 50 ml of medium and sealed with butyl rubber stoppers. The media-containing serum bottles were degassed and flushed with H_2_:CO_2_ (80%:20%), reduced with 0.5 mM cysteine-HCl, supplemented with 1.3 mM FeCl_2_ · 2H_2_O, and the headspace of each bottle was filled with 200 kPa of H_2_:CO_2_. Each growth medium was inoculated in triplicate with a 1% (vol vol^−1^, ~10^5^ cells ml^−1^) logarithmic growth-phase culture that had been transferred at least three times on that terminal electron acceptor. Cultures were incubated at 55°C until they reached the late logarithmic-to-early stationary growth phase (~5 days). For cell counts, samples were preserved using 2% (vol vol^−1^) formaldehyde and mixed with an oxalate solution (28 g L^−1^ ammonium oxalate and 15 g L^−1^ oxalic acid) to dissolve the particulate iron. Cells were filtered using 0.2-μm pore size black polycarbonate membrane filters (Whatman, Little Chalfont, UK), stained with 0.1% (wt vol^−1^) acridine orange for 3 min, and counted using epifluorescence microscopy (Hobbie et al., 1977).

In addition to the biotic samples (heat + cells), there was an uninoculated control (heat/no cells) for each Fe(III) (oxyhydr)oxide mineral that was incubated at the same temperature and duration as the biotic samples and an uninoculated control that was incubated at room temperature (no heat/no cells). The composition of the controls was otherwise identical to the biotic samples. These two controls served to determine the abiotic effect of growth medium alone and growth medium plus incubation temperature on the minerals present and thus to differentiate microbially induced mineral transformations from abiotic mineral transformations. Fe(II) concentrations were measured spectrophotometrically for all samples following dissolution in oxalate solution using the ferrozine assay (Phillips and Lovley, 1987). Each sample was unfiltered and filtered through a 0.22-μm pore size polyethersulfone (PES) membrane syringe filter (Corning Inc., Corning, New York, USA) prior to dissolution in the oxalate solution to determine the proportion of Fe(II) that was in the microparticulate form.

### Mineral analyses

For spectroscopy and XRD analyses, each sample was filtered through a 0.2-μm pore size polycarbonate filter (Whatman, Little Chalfont, UK) in an anoxic chamber, dried for at least 12 h, and stored in an anoxic chamber to minimize oxygen exposure and mineral alteration.

Visible-near infrared (VNIR) spectra were collected using an ASD Fieldspec 4 Max spectrometer in bidirectional geometry (input = 30°, emission = 0°) with an Ocean Optics HL-2000 light source directed down toward a 1,000-μm Si optical fiber and an 8° foreoptic for collection. The samples were loaded into a matte black sample cup in the air and leveled without packing. The samples were in the air for ~5 min. Each spectrum had an average of 3 × 240 136 ms integrations and was referenced to a Spectralon. Continuum removal was performed manually using OPUS software (Bruker Optics). Fourier transform infrared attenuated total reflectance (FTIR-ATR) spectra were obtained on a Bruker ALPHA FTIR spectrometer with a platinum diamond ATR attachment placed inside an anoxic chamber. Spectra were collected from 360–4,000 cm^−1^ at 8 cm^−1^ resolution. Each spectrum had an average of 128 scans. Continuum removal was performed with OPUS software using a concave rubberband correction.

Mössbauer spectroscopy was performed on anoxically mounted dried powder samples that were ground lightly with sugar and secured in plastic washers backed with Kapton tapes. Spectra were collected at Mount Holyoke College on a Web Research (now See-Co) W302 Mössbauer spectrometer at 295, 220, 150, 80, and 4 K (starting with the lowest temperature) employing a Janus closed cycle He compressor at <295 K. Samples were transported under an inert atmosphere to Mount Holyoke College and loaded into the sample tube, which was immediately purged and filled with He gas. Each 1024-channel spectrum was folded about the midpoint and calibrated to an α-Fe foil using the WMOSS4 program. Spectra were fit using the Mexfield program, provided by Eddie DeGrave and Toon Van Alboom of the University of Ghent, Belgium. The Mexfield program uses Lorentzian line shapes and solves the full hyperfine interaction Hamiltonian to minimize the chi-squared deviation between the data and the model using center shift, quadrupole splitting, linewidth, ±hyperfine field, and field broadening as free parameters (Vandenberghe et al., 1994).

X-ray diffraction (XRD) analysis was performed using a Rigaku Smartlab II SE XRD (Cu Kα radiation; Bragg-Brentano geometry). Anoxically dried samples were transported in an inert atmosphere to the XRD facility. XRD patterns were acquired in the air (42-min scans) on zero-background sample holders from 5 to 80° 2θ with a 0.02° step size at a rate of 2° per minute. Baseline corrections and mineral identifications used the Rigaku Smartlab II software (ICSD PDF2 database; 2019 version).

Transmission electron microscopy (TEM) was performed in the Materials Characterization and Processing Center at Johns Hopkins University. It was performed on a Thermo Fisher TF30 instrument operating at 300 kV with an EDAX windowless silicon drift energy dispersive X-ray (EDX) detector. Conventional imaging, high-resolution lattice imaging (HRTEM), selected area electron diffraction (SAED), scanning transmission electron microscopy (STEM), bright-field (BF) and high-angle annular dark-field (HAADF) imaging, and STEM EDX maps were collected for each bioreacted and heated control mineral sample. Samples analyzed by TEM were shipped cold to Johns Hopkins in their original media, and a grid was loaded with samples directly from the bottles onto lacey carbon grids.

## Results

### Cell growth on ferrihydrite

**Figure 1:**
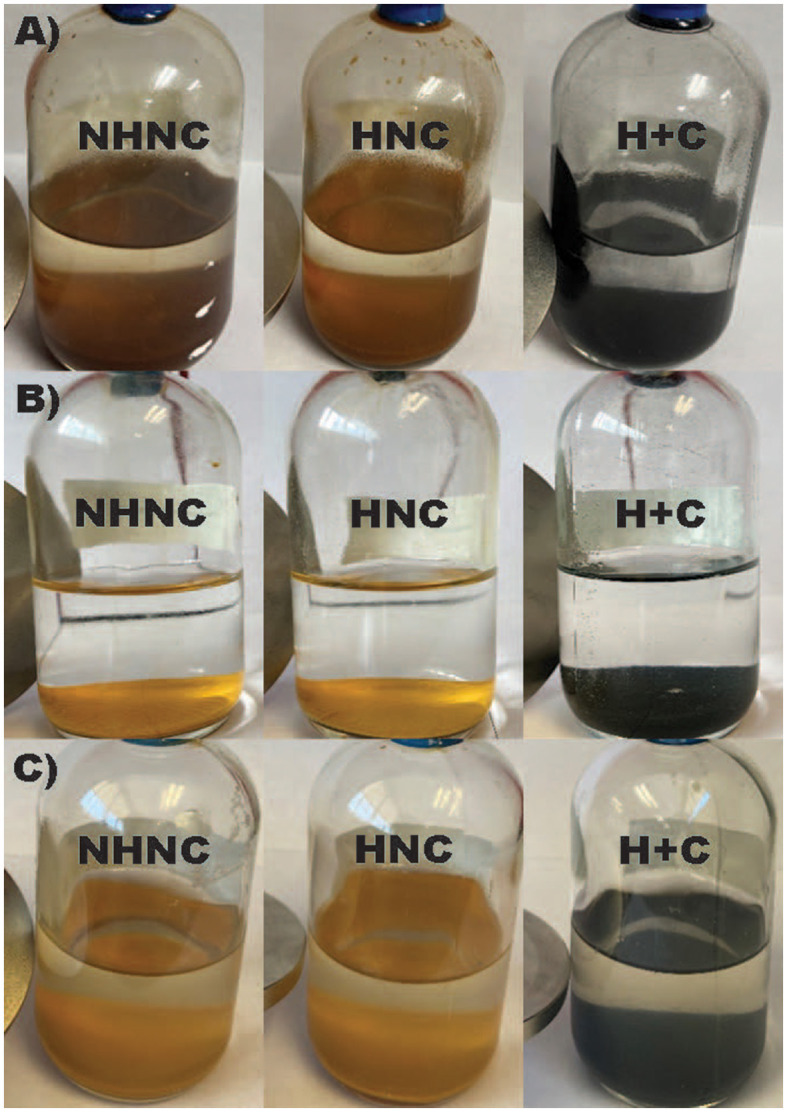
Images of *Desulfovulcanus ferrireducens* grown on ferrihydrite **(A)**, lepidocrocite **(B)**, and akaganeite **(C)**. From left to right, the incubations are unheated abiotic controls (no heat/no cell, NHNC), heated abiotic controls (heat/no cell, HNC), and the biotic samples (heat + cells, H + C). A magnet at the bottom left of each bottle indicates a magnetic product.

*Desulfovulcanus ferrireducens* grew to ~10^7^ cells ml^−1^ when grown on ferrihydrite with 24 mM of Fe^2+^ in the medium. A separate filtration step through a 0.2-μm pore size filter prior to the addition of oxalate solution showed that 84% of the biogenic iron product was retained by the filter. The iron produced was visibly black and magnetic (Figure 1A). The concentrations of Fe^2+^ in the heated and unheated abiotic controls were 2–3 mM, there was no visible color change to the iron (Figure 1A), and neither sample was magnetic.

**Figure 2:**
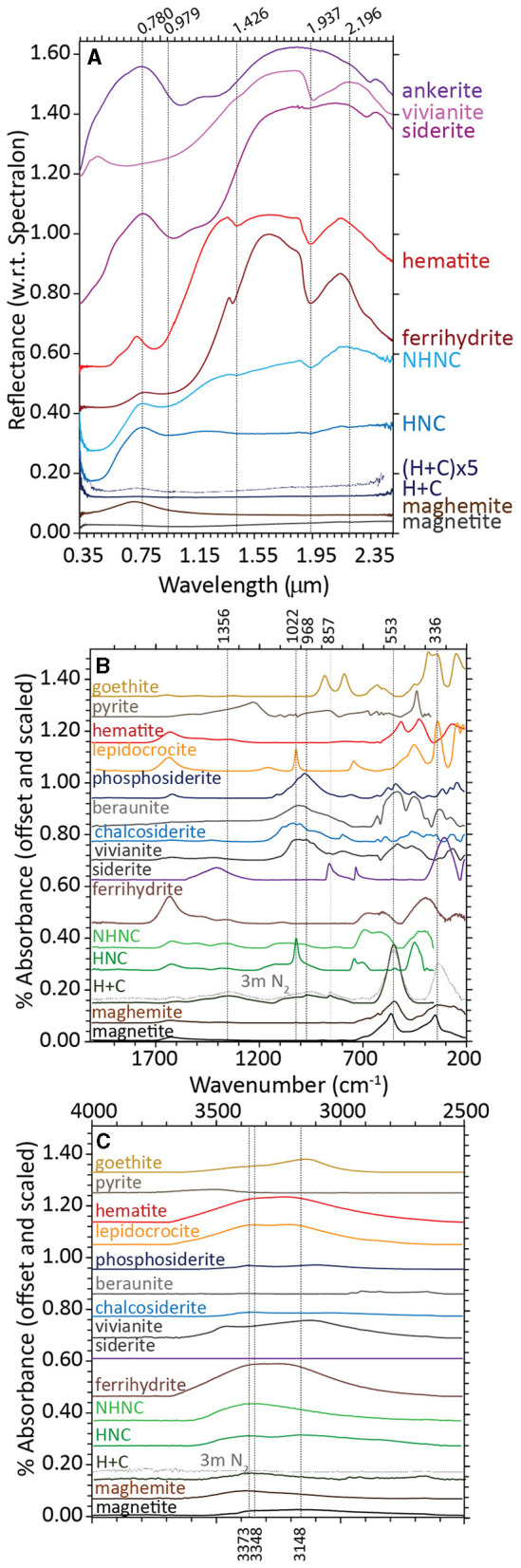
Visible-near infrared [VNIR, **(A)**] and Fourier-transform infrared attenuated total reflectance [FTIR-ATR, **(B, C)**] spectra for biotic (heat + cells), heated abiotic (heat/no cells), and unheated abiotic (no heat/no cells) mineral transformations of ferrihydrite for *Desulfovulcanus ferrireducens* along with iron mineral reference standards. All spectra are offset for clarity.

Ferrihydrite that was bioreduced by *D. ferrireducens* is spectrally flat and minimally reflective in its VNIR spectrum, indicating the presence of a highly absorbing and opaque phase like magnetite (Figure 2A). When the spectrum is vertically stretched, a maximum wavelength of 0.743 μm is observable, which could indicate a cation-deficient magnetite or the mixing of multiple iron-bearing phases. The VNIR spectra for the heated and unheated controls are muted relative to the parent ferrihydrite spectrum, but they retain the visible wavelength maximum of 0.78 μm, almost identical to that in ferrihydrite. The hydration-related absorption features are visible but partially shifted to 0.915 and 1.937 μm for the unheated control and 0.979 μm, 1.937 μm, and 2.196 μm for the heated control in uncorrected spectra. Continuum-removed peak positions, while dependent upon the subjective choice of continuum anchor points, can often offer more direct comparisons between absorption features. Supplementary Figure 1, Supplementary Table 2 examine the standard and continuum-removed VNIR spectra more closely, and both uncorrected and continuum-removed VNIR data and references are included in the Supplementary material.

The FTIR-ATR spectra of ferrihydrite bioreduced by *D. ferrireducens* completely lose the ferrihydrite-like broad doublet at 672 and 603 cm^−1^ and broad absorption at 401 cm^−1^ (Figure 2B). These are replaced by a large, symmetric absorption at 553 cm^−1^ and a broad, asymmetric absorption at 336 cm^−1^. The shape and position of these features are consistent with a cation-deficient magnetite. The heated control spectrum also loses most of the ferrihydrite-like absorptions but instead indicates a transformation to lepidocrocite, which is identifiable by a sharp absorption at 1,022 cm^−1^ (Sklute et al., 2018). The unheated control spectrum resembles ferrihydrite at frequencies of <800 cm^−1^.

At higher frequencies, a broad triplet is visible, spanning from ~970 to 1,117 cm^−1^, likely due to the absorption of various media-related compounds, such as phosphate, sulfate, or organics, to the mineral surface (c.f., Arai and Sparks, 2001). These features are in a similar location but more pronounced in the heated control spectrum. In the bioreduced sample spectrum, the complex feature is shifted to lower wavenumbers and includes a sharp embedded feature at ~852 cm^−1^. Combined with a broad absorption centered at ~1,356 cm^−1^, an absorption of 852 cm^−1^ is a good indicator of carbonate, although both features are shifted to lower frequencies than those typically observed for siderite. Instead, these features are similar to those reported for carbonate green rust (Antony et al., 2008), which can form in circumneutral solutions of similar composition (Schwertmann and Fetcher, 1994). The shift in the underlying broad feature centered near 968 cm^−1^ may point to the absorption of cell-associated phosphate or aqueous phosphate. Iron phosphate minerals contain additional absorptions at low wavenumbers that are not observed in this spectrum. Whatever the source of these absorptions, their shape and position in the bioreduced sample spectrum are clearly distinct from those in the control spectra. At higher wavenumbers (Figure 2C), the hydration feature position is closer to that of maghemite than magnetite, confirming that the spinel is likely some intermediate or cation-deficient magnetite, even under the reducing conditions of the study.

**Figure 3:**
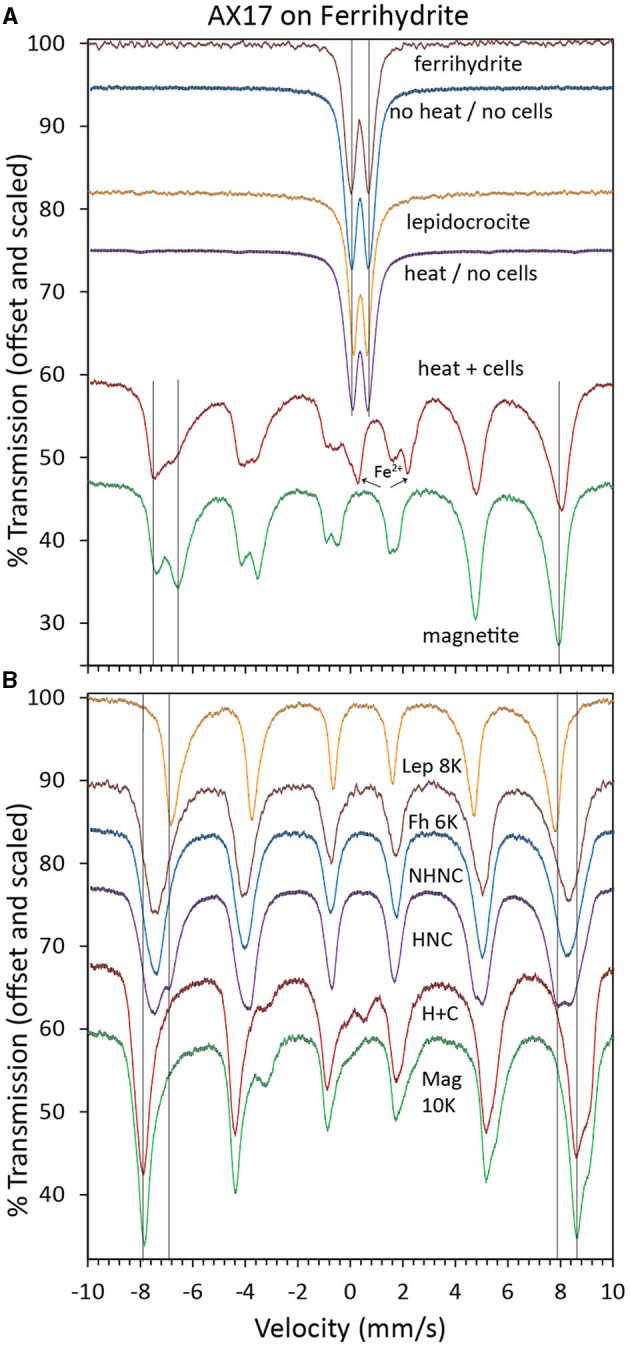
Mössbauer spectra at 295K **(A)** and 4K **(B)** of biotic (heat + cells), heated abiotic (heat/no cells), and unheated abiotic (no heat/no cells) mineral transformations of ferrihydrite for *Desulfovulcanus ferrireducens* along with iron mineral reference standards. Each spectrum is shown along with its associated fits and parameters, which are color-coded, scaled, and offset for clarity.

The 295K Mössbauer spectrum of the bioreduced sample is a mixture of sextets and doublets, including a prominent ferrous doublet (Figure 3A). The sextet structure is similar to that of cation-deficient nanophase magnetite, but admixing with other phases cannot be ruled out. The 4K spectrum confirms that the sample is not pure magnetite (Figure 3B). The lingering doublet structure centered near 0.2 mm/s, increased the depth to the shoulder at 9 mm/s, and decreased the absorption depth near −3.3 mm/s and 2.7 mm/s all point to a mixture of phases or a modification to the magnetite structure. There was too much overlap in the central region to identify minor green rust. The 295K spectra of the control samples were doublets with parameters consistent with ferrihydrite or lepidocrocite (Figure 3A). Mössbauer spectroscopy cannot distinguish these two minerals at 295K. At 4K, the heated control spectrum shows a clear mixture of ferrihydrite-like and lepidocrocite-like sextets, while the unheated control spectrum is almost identical to ferrihydrite (Figure 3B). Additional Mössbauer spectra for analyses at other temperatures are found in Supplementary Figures S2–S4.

Unlike the hyperthermophilic bioreduction products reported in Kashyap et al. (2023), the bulk XRD for ferrihydrite bioreduced by *D. ferrireducens* shows clear evidence of magnetite (or maghemite or cation-deficient magnetite). The broad peaks indicate small scattering domains, similar to the 11 nm magnetite reported in Sklute et al. (2018) (Supplementary Figure S5). Additional reflections are consistent with lepidocrocite, NaCl, and a small amount of non-iron carbonate, such as rhodocrocite (MnCO_3_), although rhodocrocite is unlikely given the media composition. The carbonate identified is unlikely to have been sourced from impurities in media components, is more likely a structurally similar material with a different composition, and does not appear in the library. Interestingly, the phase does not appear in the XRD of the control samples. The XRD pattern of the heated control shows clear evidence of lepidocrocite observed by FTIR-ATR. Interestingly, there is a broad swell in the baseline in the position of the magnetite/maghemite reflection of ~35.5 2θ; magnetite may be a minor product in the heated abiotic sample. The major difference between the heated abiotic sample and the bioreduced sample is in the ratio of magnetite to lepidocrocite.

**Figure 4:**
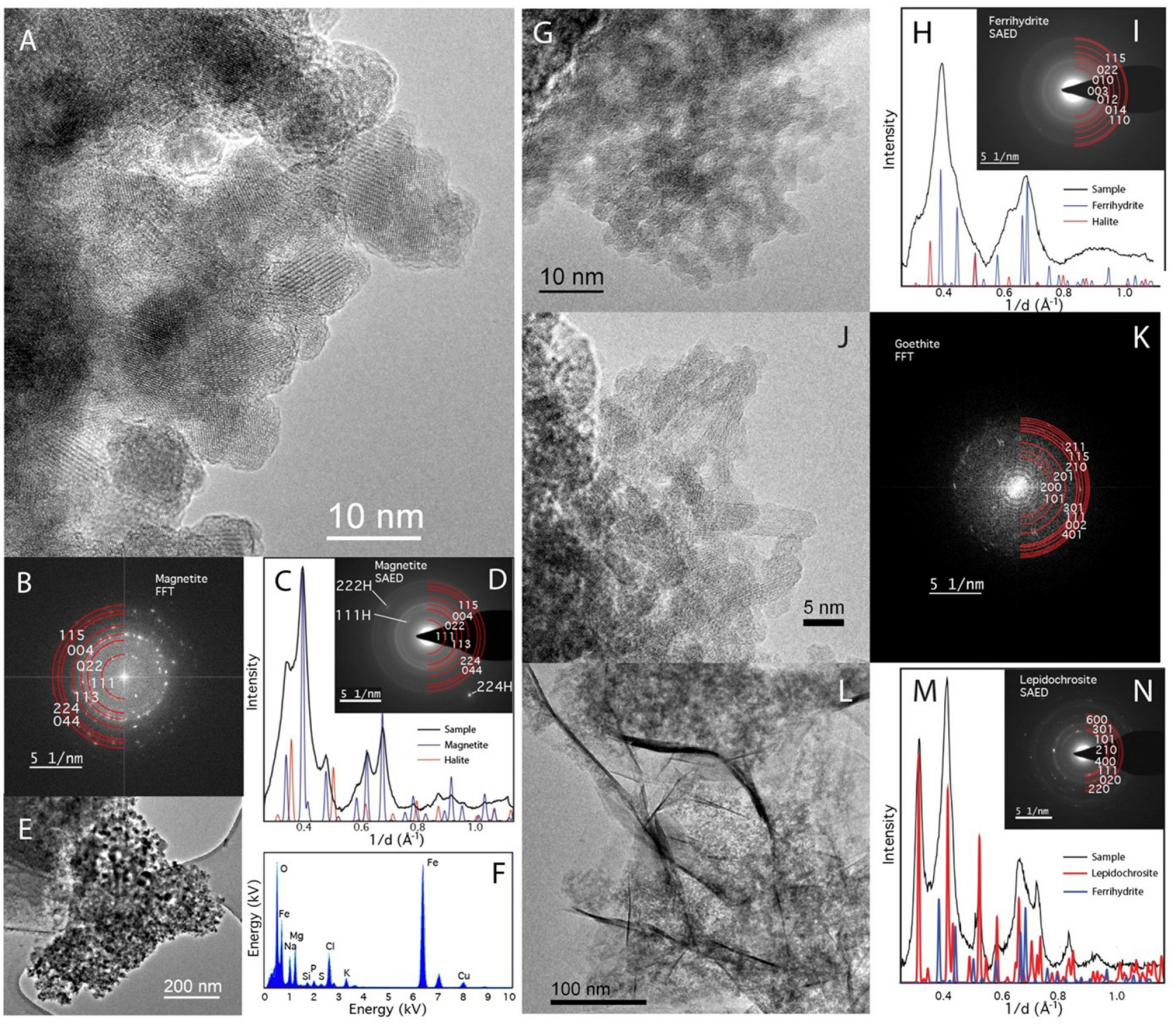
TEM of *Desulfovulcanus ferrireducens* grown on ferrihydrite. The heat + cells sample is shown in **(A–F)**, and the heat/no cells sample is shown in **(G–N)**. HRTEM showing the crystalline nature of the heat + cells sample **(A)**. The atomic layers are clearly visible in these nanophase particles. **(B)** Fast Fourier Transformation (FFT) of **(A)** blocky nanomagnetite showing clearly defined reflections for magnetite. **(C)** A radially averaged pattern of the SAED pattern in **(D)** with peaks consistent with magnetite and halite. Low magnification image of nanomagnetite aggregate **(E)**. **(F)** EDX analysis of **(E)** shows that they are a combination of Fe-rich oxide and salt (NaMg-chloride). TEM of the heated control sample showed a less clear structure **(G)**, consistent with unaltered ferrihydrite. **(H)** A radially averaged pattern of the SAED pattern in **(G)** shows poorly crystalline nanoparticles consistent with a mixture of ferrihydrite and halite. Laths of goethite found elsewhere in the sample **(I)**. **(K)** An FFT of **(J)** can be indexed as goethite. Sheets are also present **(L)**. Sheets index as lepidocrocite but are also mixed with ferrihydrite **(M, N)**.

TEM with SAED shows that ferrihydrite bioreduced by *D. ferrireducens* comprises clumps of dark, nanocrystalline but well-ordered irregular spheres, many with amorphous rims (Figures 4A, E). SAED patterns (Figures 4B–D) are consistent with a mixture of ferrihydrite, magnetite/maghemite, and halite, and EDX analysis confirms the presence of the oxide, salt, and some media components (S, P) either in or on the grains (Figure 4F). The heated abiotic control is also largely crystalline (Figures 4G–N), with clear sheets of lepidocrocite (Figures 4L–N, seen in other techniques) and laths of goethite (Figures 4J, K, not seen by any other spectroscopy). This sample also appears to have a wealth of Cu-rich spheres that cannot be explained by the experimental conditions and may be copper mobilized from the grid. These spheres are not observed in the bioreduced sample for this mineral.

### Cell growth on akaganeite and lepidocrocite

*Desulfovulcanus ferrireducens* reached a density of 2–3 × 10^7^ cells ml^−1^ when cultured separately with akaganeite and lepidocrocite. For growth on lepidocrocite, the mineral product was visibly greenish black but not magnetic (Figure 1B). The mineral product following growth on akaganeite was visibly black but not magnetic (Figure 1C). There was no visible color change to either akaganeite or lepidocrocite in either the heated or unheated abiotic controls (Figures 1B, C). The concentration of oxalate-soluble Fe^2+^ in the biotic samples was not significantly different than that of the abiotic controls, suggesting that oxalate was ineffective at dissolving the Fe^2+^ in these samples. A separate filtration step through a 0.2-μm pore size filter prior to the addition of oxalate solution showed that 82% of the biogenic iron product was retained by the filter. Oxalate is less effective at dissolving more highly crystalline phases (Schwertmann and Fetcher, 1994). The lack of oxalate-soluble Fe^2+^ does not necessarily indicate that Fe^2+^-bearing mineral phases were not produced. Mössbauer spectroscopy clarified the extent of mineral reduction in these samples.

**Figure 5:**
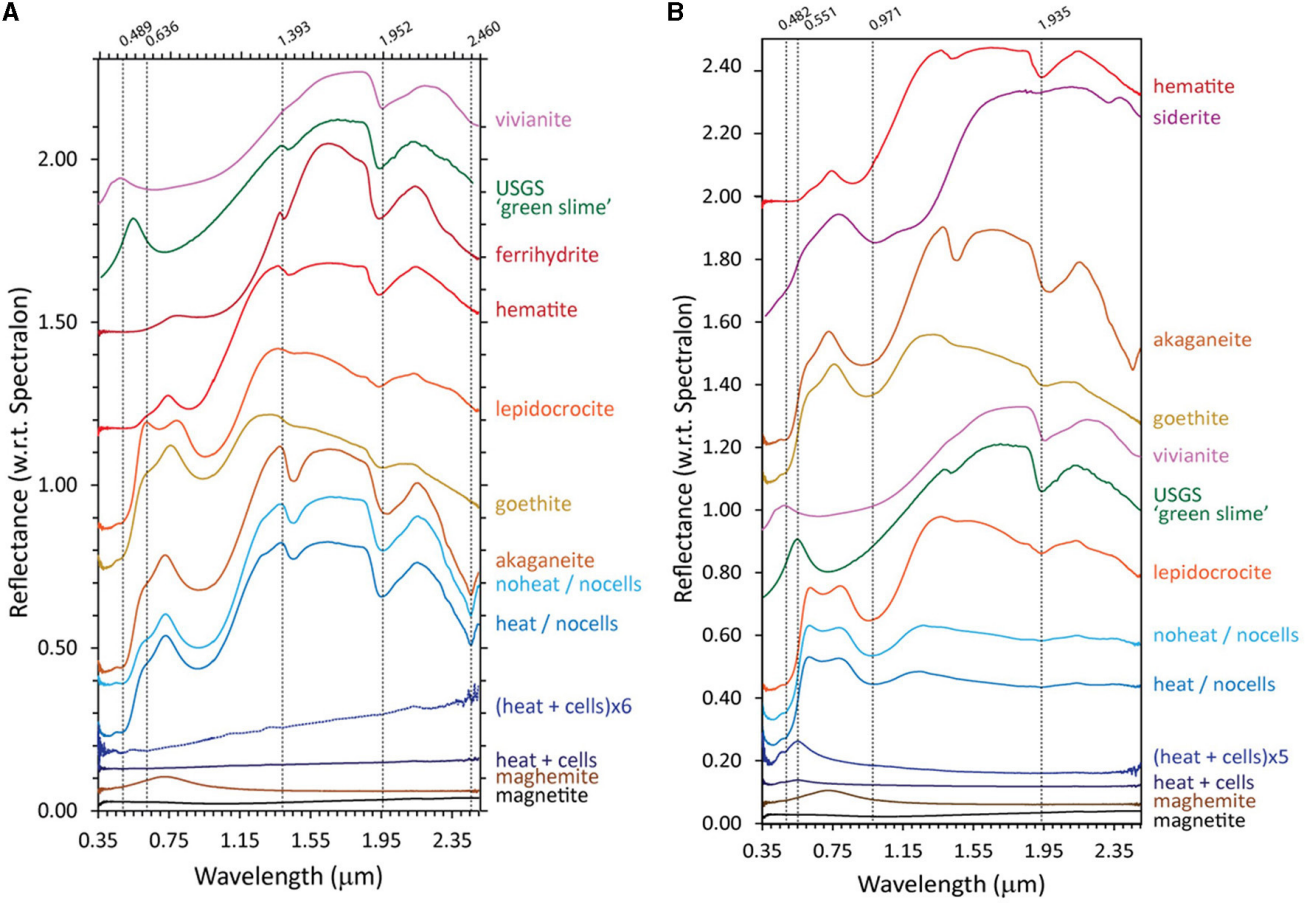
Visible-near infrared (VNIR) spectra for biotic (heat + cells), heated abiotic (heat/no cells), and unheated abiotic (no heat/no cells) mineral transformations of akaganeite **(A)** and lepidocrocite **(B)** for *Desulfovulcanus ferrireducens* along with iron mineral reference standards. All spectra are offset for clarity.

Like ferrihydrite, the akaganeite bioreduced by *D. ferrireducens* is spectrally flat and minimally reflective in its VNIR spectrum, indicating the presence of a highly absorbing and opaque phase (Figure 5A). However, unlike ferrihydrite, when the VNIR spectrum is stretched vertically, it does not display a maghemite-like VIS maximum but rather a red slope. It also lacks the distinctive akaganeite absorption at 2.460 μm. The heated and unheated abiotic control spectra are nearly identical to the parent mineral spectrum. Lepidocrocite bioreduced by *D. ferrireducens* is also spectrally flat and minimally reflective in its VNIR spectrum (Figure 5B). When the VNIR spectrum is stretched vertically, it has a clear VIS maximum at 0.551 μm, which is in the same position as the VIS maximum in a natural green rust sample (USGS “green slime”). Retroactive examination of the VNIR spectrum reported for the bioreduction of lepidocrocite by the marine hyperthermophile *P. delaneyi* (Kashyap et al., 2023) revealed a similar VIS maximum upon spectrum expansion. Supplementary Figures S6, S7 and Supplementary Table S2 examine the standard and continuum-removed VNIR spectra more closely, and both uncorrected and continuum-removed VNIR data and references are included in the Supplementary material.

**Figure 6:**
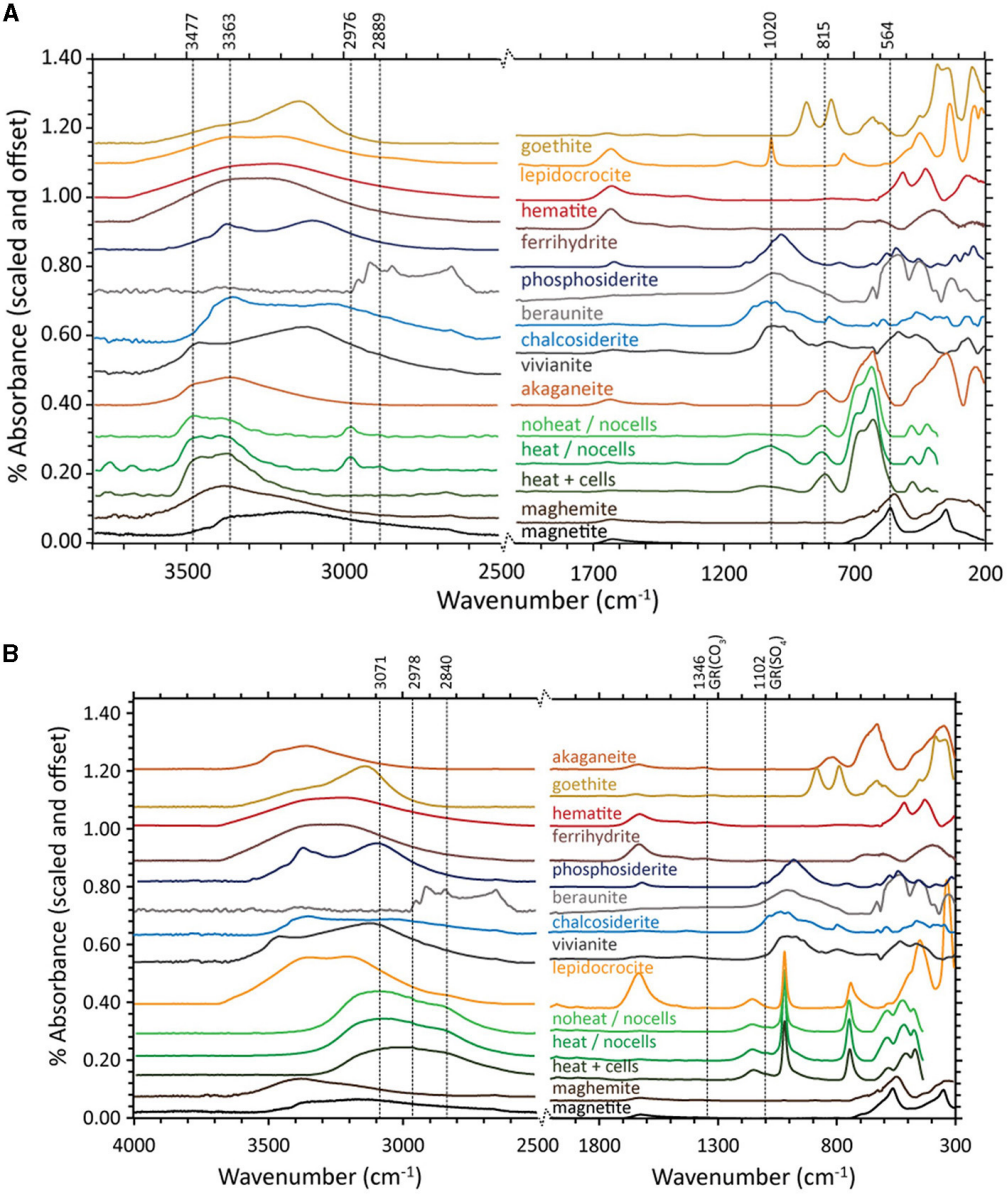
Fourier-transform infrared attenuated total reflectance (FTIR-ATR) spectra for biotic (heat + cells), heated abiotic (heat/no cells), and unheated abiotic (no heat/no cells) mineral transformations of akaganeite **(A)** and lepidocrocite **(B)** for *Desulfovulcanus ferrireducens* along with iron mineral reference standards. All spectra are offset for clarity.

The FTIR-ATR spectra of akaganeite that was bioreduced by *D. ferrireducens* and the two control spectra (Figure 6A) are similar in the ~900–500 cm^−1^ range typically used to identify that mineral (Sklute et al., 2018). There is a slight shoulder in the bioreduced spectrum near 564 cm^−1^ and a decrease in the feature near 420 cm^−1^ that could indicate trace magnetite. While continuum removal can affect the latter, changes in this feature shape can be observed in additional experiments where lower wavenumber range coverage is available. All three experimental spectra display separation of the peaks that make up the main akaganeite asymmetric absorption at ~626 cm^−1^. The bioreduced sample, however, has increased area in the higher wavenumber peak of ~681 cm^−1^ with respect to the peak ~624 cm^−1^. Although digital reference spectra for sulfate green rust are not available, this increase in intensity is consistent with the peak position for sulfate green rust reported by Misawa et al. (1969). Admixing with the other major green rust feature shown in the abovementioned study near 788 cm^−1^ could be responsible for the slight shift to lower wavenumbers of the akaganeite peak near 805 cm^−1^ (typically a doublet centered near 827 cm^−1^). However, more recent studies of green rust and green rust oxidation products by Antony et al. (2008) report many absorptions not observed in our samples. Their chloride green rust matches our sample and is more realistic for the solution chemistry. Both the bioreduced and heated control samples show absorptions in the phosphate/sulfate region near 1,020 cm^−1^; the shape and location of these features differ between the two samples and are much more intense in the heated control. Both control samples also have features near 2,976 and 2,889 cm^−1^, which are consistent with organics that are added to the media. Of note is the absence of these features in the bioreduced sample, indicating these elements are consumed during bioreduction.

FTIR-ATR spectra of lepidocrocite bioreduced by *D. ferrireducens* and the two control spectra (Figure 6B) are almost identical to the reference lepidocrocite is at <1,200 cm^−1^. Above 1,200 cm^−1^, all three experimental spectra differ from the reference, primarily due to the loss of hydration features approximately at 1,623 cm^−1^ and >3,250 cm^−1^. All three spectra share a muted, broad feature from ~2,590 to 3,290 cm^−1^. While this range overlaps with the lipid region reported in biofilm spectral studies (c.f., Gieroba et al., 2020), this seems more likely due to tightly bound water that survived anaerobic drying. A small feature in the two control spectra near 1,089 cm^−1^ could indicate a carbonate, but it is too small to match any specific phase and is likely an adsorption product. This feature is, interestingly, less intense in the bioreduced spectrum. No spectral evidence in this wavelength range explains the color change observed in the VNIR.

**Figure 7:**
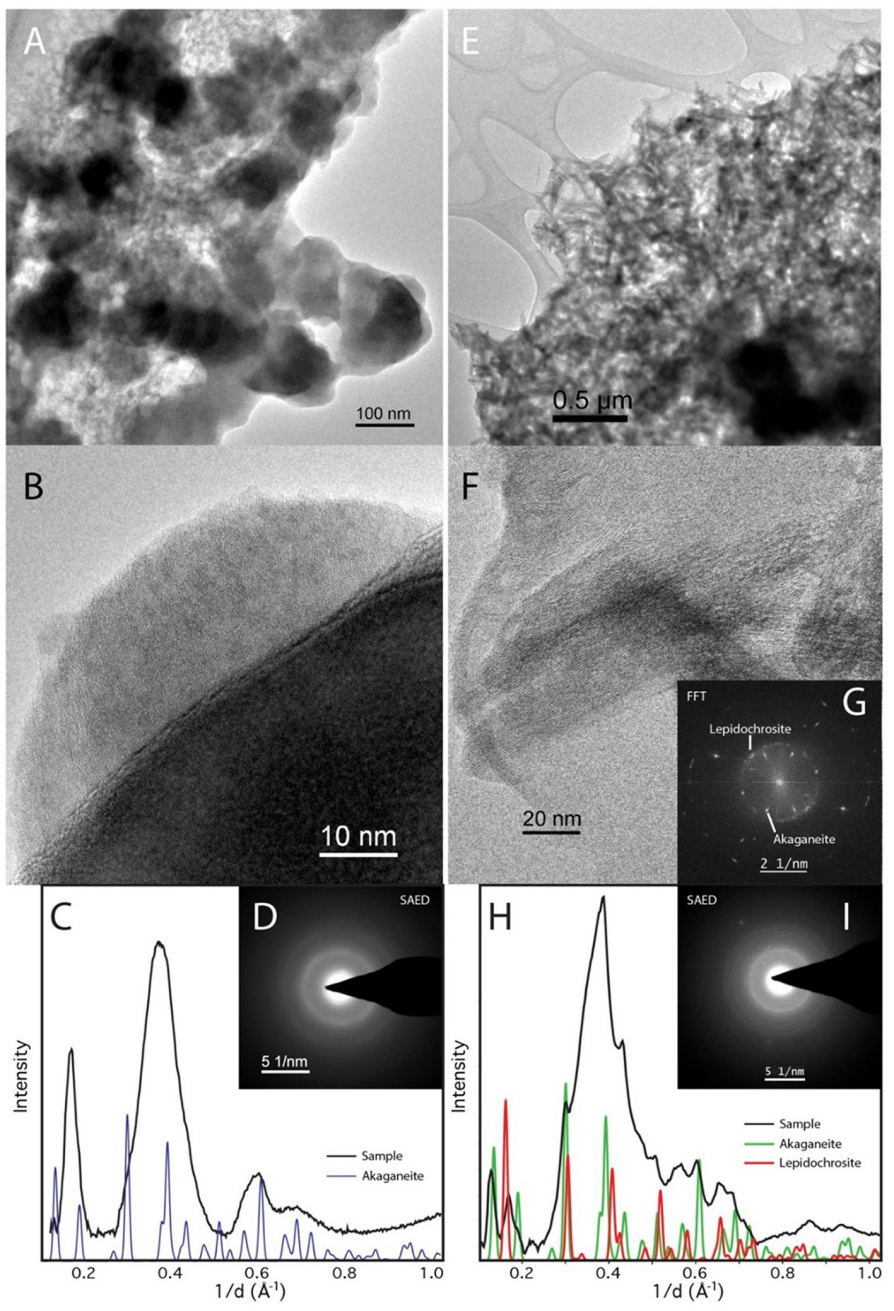
TEM of *Desulfovulcanus ferrireducens* grown on akaganeite. The heat + cells sample is shown in **(A–D)**, and the heat/no cells sample is shown in **(E–I)**. Amorphous Fe-rich nanospheres (AFNS) are ~100 nm in diameter **(A)**. High magnification image of AFNS illustrating their amorphous nature **(B)**. The AFNS produce broad rings consistent with their amorphous state **(C, D)**. Aggregate of akaganeite laths **(E)**. HRTEM image of akaganeite laths and curved structure consistent with lepidocrocite **(F)**. **(G)** Fast Fourier Transformation (FFT) of **(F)** The ring comes from curled lepidocrocite. SAED patterns of mainly akaganeite and some lepidocrocite **(H, I)**.

Mössbauer spectra of akaganeite bioreacted by *D*. *ferrireducens* and control spectra (Supplementary Figures S8A, B) show subtle changes in the iron in the sample. Temperature series and fit parameters (Supplementary Figures S9–S11) show that the heated control sample has a new ferric phase and that the bioreacted sample has new ferric and ferrous phases, both of which persist below the magnetic ordering temperature of the ferric phases of the unheated control sample. The fit parameters for the new phase in the bioreacted sample are not unique due to overlap with other phases, but the general spectral shape suggests that admixing with green rust or a structurally and energetically similar phase is possible (Supplementary Figure S8). The SAED of the TEM sample is consistent with a mixture of akaganeite and lepidocrocite (Figure 7H). While the lepidocrocite would explain a new ferric doublet, the Mössbauer doublet for lepidocrocite should persist below 80K, which is not observed for this new phase.

Mössbauer spectra of lepidocrocite bioreacted by *ferrireducens* and control spectra (Supplementary Figures S12A, B) are almost identical to the reference lepidocrocite spectrum. Temperature series and fit parameters (Supplementary Figures S13–S15) also fail to identify any changes to the iron in these samples that would account for the color changes recorded in the VNIR. However, it is possible that small amounts of green rust could be hidden within the broad lepidocrocite features (Supplementary Figure S12).

Bulk XRD spectra of akaganeite bioreduced by *D*. *ferrireducens* and control spectra are almost identical (Supplementary Figure S16) and display a mixture of akaganeite with NaCl. An additional reflection near 13.97 2θ in the heated control spectrum is consistent with a reflection for nanophase lepidocrocite, as reported by Sklute et al. (2018). In that sample, the remaining lepidocrocite reflections align with slight baseline swells or are obscured by other peaks. For the bioreduced sample, there is a unique reflection of ~13.16 2θ. This is the position of the main reflection in the iron phosphate vivianite. While a single reflection is insufficient for phase identification, when added to the fit, the program Match! determined that it comprises >14 mol% of the sample. This could account for the color change observed in the VNIR. However, at such concentrations, the feature should also be visible in both FTIR-ATR and Mössbauer spectra. Vivianite features in Mössbauer and FTIR spectra could potentially be obscured by absorptions of other phases, but a more likely scenario is that the XRD match percentage is driven by the XRD baseline swell due to the fine-grained nature of the sample.

TEM with SAED of akaganeite samples shows that the heated control comprises 100–200 × 25 nm akaganeite laths, whose SAED indicates a structural shift toward lepidocrocite. The bioreduced sample, on the other hand, is comprised of poorly crystalline 50–200 nm balls (Figure 7) that we hereafter refer to as amorphous iron-rich nanospheres (AFNS). This sample also contains a film not observed in the heated control that could be the result of biological activity.

**Figure 8:**
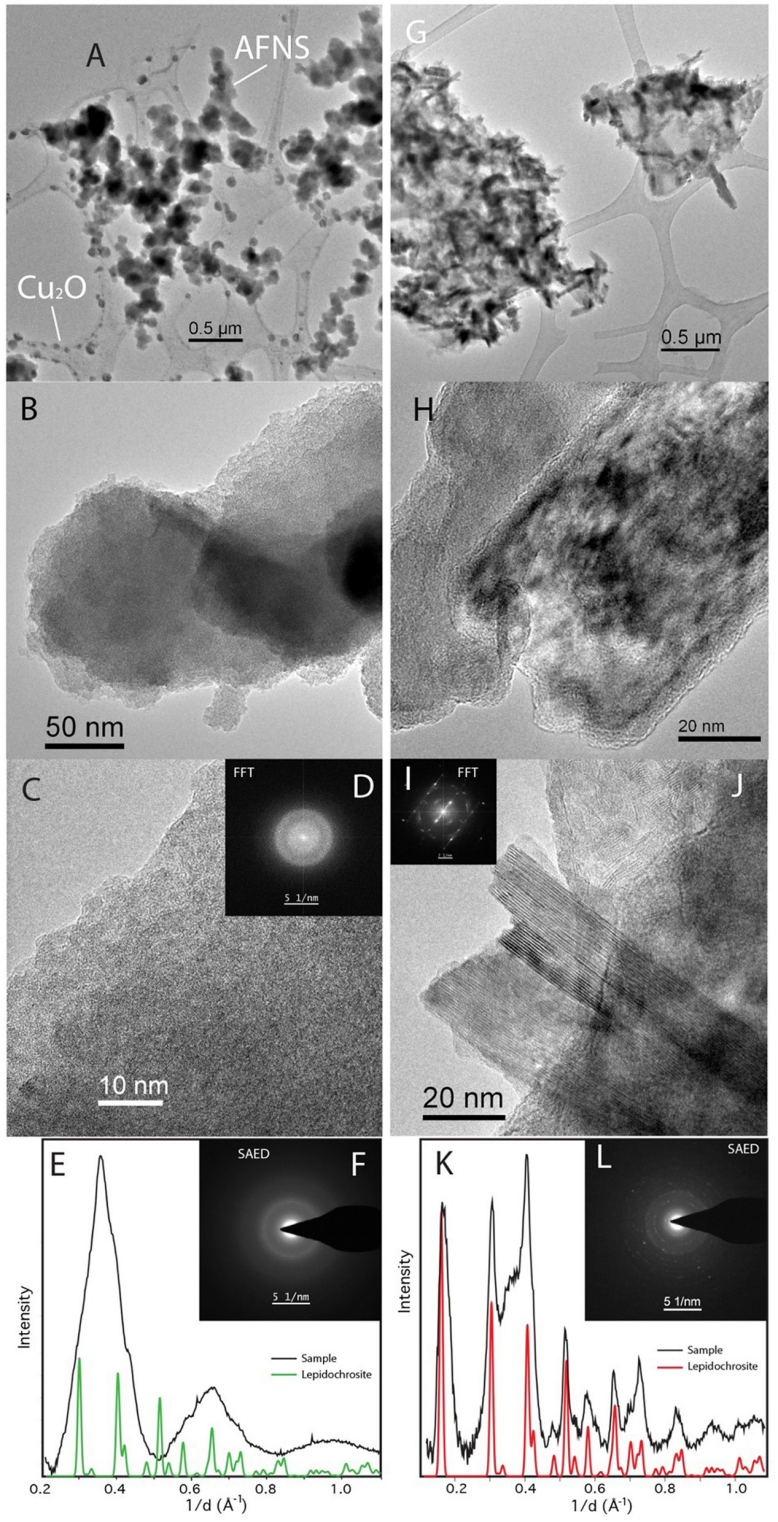
TEM of *Desulfovulcanus ferrireducens* grown on lepidocrocite. The heat + cells sample is shown in **(A–F)**, and the heat/no cells sample is shown in **(G–L)**. Amorphous Fe-rich nanospheres (AFNS) along with Cu_2_O nanospheres **(A)**. Close-up image of AFNS illustrating their amorphous nature **(B, C)**. **(D)** Fast Fourier Transformation (FFT) of **(C)** showing broad diffuse rings indicative of amorphous material. SAED patterns of diffuse rings from AFNS **(E, F)**. Laths of lepidocrocite **(G)**. HRTEM image of laths that are rimmed by amorphous material **(H)**. HRTEM of well-formed lepidocrocite exhibiting the 6.2 Å spacing **(I, J)**. SAED patterns of lepidocrocite have distinct reflections **(K, L)**. The plateau between the second and third peaks is associated with amorphous material found mixed with the lepidocrocite laths.

Bulk XRD was not possible for the lepidocrocite experiments, but TEM with SAED (Figure 8) shows that the heat-treated control sample is largely crystalline, with the laths displaying amorphous rims not uncommon in synthetic nanophase iron (hydro)oxides. The bioreduced sample, however, is largely amorphous, similar to the akaganeite experiments. However, unlike the akaganeite bioreduced sample, these spheres have jagged rather than smooth edges (Figure 8B). While the AFNS on both akaganeite and lepidocrocite are amorphous, assuming they are the same material or metamaterial would be a mistake. The AFNS in this sample is also accompanied by Cu spheres of unknown origin.

The presence of Cu spheres under different experimental conditions (heated control in the ferrihydrite experiments vs. bioreduced in the lepidocrocite experiments) suggests that they are analytical contaminants rather than experimental products and that the mobilization of Cu is related to specific solution chemistry macroenvironments or microenvironments in these two experiments.

## Discussion

Methanogens, sulfur reducers, and nitrate reducers are the anaerobic bacteria and archaea most studied and commonly associated with deep-sea hydrothermal vents (Huber and Holden, 2006; Holden et al., 2012; Sievert and Vetriani, 2012). Thermophilic iron-reducing bacteria and archaea are also found at deep-sea hydrothermal vents (Slobodkin et al., 2001; Kashefi et al., 2002, 2003; Slobodkina et al., 2009a,b, 2012; Ver Eecke et al., 2009; Lin et al., 2014; Kashyap et al., 2022), but little is known about them. Examining thermophilic iron reducers from vent environments, their mineral requirements, and their mineral products will expand our understanding of microbial community dynamics and their biogeochemical effect on hydrothermal vent sites. Ferrihydrite is the predominant form of iron in anaerobic, non-sulfidic environments (Lovley, 1991), but other more crystalline Fe(III) (oxyhydr)oxides can be present as well. In sulfide chimneys and seafloor sulfide deposits at hydrothermal vents, two-line ferrihydrite, six-line ferrihydrite, lepidocrocite, akaganeite, goethite, and biogenic Fe(II) (oxyhydr)oxide have been identified (Lin et al., 2016b; Toner et al., 2016). As observed for *D. ferrireducens* and *P. delaneyi*, which were both isolated from hydrothermal vent sulfide chimneys (Lin et al., 2016a; Kashyap et al., 2018, 2022), a range of synthetic nanophase Fe(III) (oxyhydr)oxide minerals serve as terminal electron acceptors for anaerobic bacteria and archaea that live within these hydrothermal deposits.

**Figure 9:**
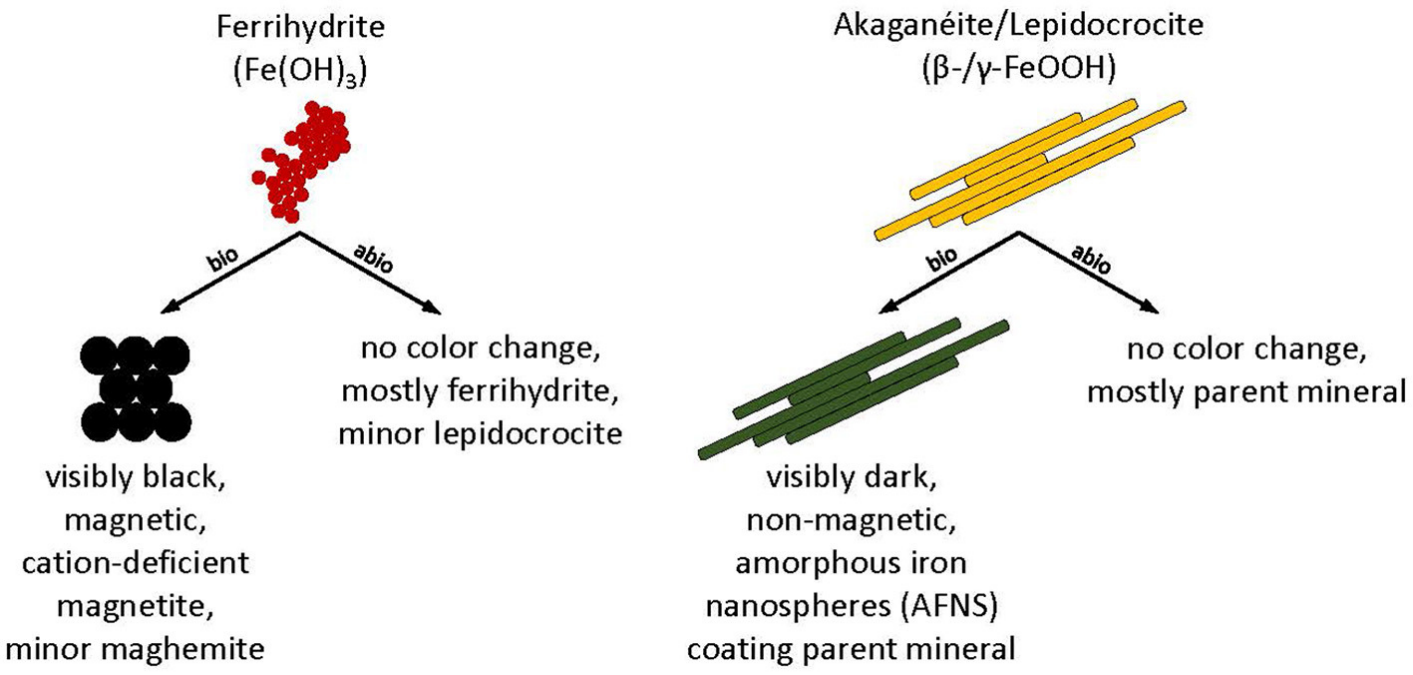
Conceptual model and summary of biotic (bio) and abiotic (abio) mineral transformations of ferrihydrite, akaganeite, and lepidocrocite that occurred in *Desulfovulcanus ferrireducens* growth media at 55°C.

In this study, *D. ferrireducens* reduced synthetic nanophase ferrihydrite to primarily cation-deficient magnetite based on VNIR, FTIR-ATR, and Mössbauer spectroscopies, XRD, and SAED (Figure 9). This was consistent with geochemical modeling predictions (Supplementary Table S1) and observations made on hyperthermophilic iron reducers (Kashefi et al., 2008; Lin et al., 2014; Kashyap et al., 2018, 2023) and mesophilic bacteria (Lovley et al., 1987; Sparks et al., 1990; Hanzlik et al., 1996; Zachara et al., 2002; Vali et al., 2004; Roh et al., 2006; Li et al., 2009; Perez-Gonzalez et al., 2010). Therefore, the bioreduction of synthetic nanophase ferrihydrite to magnetite appears to be the expected mineral product for autotrophs and heterotrophs, bacteria and archaea, and mesophiles up through hyperthermophiles. The formation of magnetite is primarily controlled by ferrous iron concentration and the supply rate (Zachara et al., 2002; Hansel et al., 2003). High Fe(II) concentrations and high supply rates result in Fe(II) sorption onto a mineral and subsequent nucleation of magnetite, but only above a threshold of Fe(II) concentration (Zachara et al., 2002; Hansel et al., 2003). However, considering biogeochemical end products, the solution chemistry cannot be ignored. For instance, Barron and Torrent (2004) showed that the presence of phosphate in solution will favor the formation of lepidocrocite over more stable phases like goethite or hematite. In crystallization experiments starting from solutions containing both Fe(II) and Fe(III), the product at high P/Fe ratios was a mixture of lepidocrocite and magnetite. Phosphate will also suppress the pathway to goethite (Barron et al., 2003), even in systems where goethite is favored, such as those containing carbonate (Schwertmann and Cornell, 2000).

Selective incorporation of organics and cations into magnetite formed from dissimilatory iron reduction has been proposed to distinguish biomagnetite from inorganic and abiotic magnetites (Jimenez-Lopez et al., 2010; Perez-Gonzalez et al., 2010, 2013; Markovski et al., 2017). Herein, the magnetite that precipitated from the bioreduction of ferrihydrite showed differences from synthetic magnetite. For example, our FTIR-ATR data showed that bioreduced magnetite has a more symmetric main peak ~553 cm^−1^ and a prominent peak ~336 cm^−1^ that is consistent in position with that in maghemite, but much sharper. The hydration feature of ~3,348 cm^−1^ is also more consistent with maghemite than magnetite. In addition to the oxide features, there are peaks consistent with carbonate (although not siderite) at ~857 and 1,356 cm^−1^. While these peaks could be due to carbonate green rust (c.f., Legrande et al., 2001), TEM did not reveal any particles consistent with green rust. There were also peaks associated with phosphate at ~1,022 and 968 cm^−1^. The phosphate features in the bioreduced magnetite are shifted in position from those of the control samples, and while they are consistent in shape and position with the abiotic samples produced in Jurado et al. (2003), in that study, P/Fe was >0.5 for the features to be visible. In our samples, P/Fe from media sources is 0.01 (0.07 g KH_2_PO_4_ per 50 ml serum bottle). At these concentrations, neither the effect of phosphate on precipitation product nor FTIR features should be prominent. These results suggest that an additional source of phosphate, possibly from the cells themselves, contributed to the final product. EDX confirmed that the magnetite samples produced by *D. ferrireducens* showed close association with other cations, and incorporating those cations into the magnetite structure cannot be ruled out. Thus, we conclude that, while it is possible to produce a similar product abiotically, it could not come from a fluid of the same composition. Put into a life detection context, if elemental analysis of the fluid along with a spectroscopic characterization of the precipitate solid showed a similar mismatch, biological activity could potentially be inferred. More experimental work would need to be performed to verify this.

*Desulfovulcanus ferrireducens* also transformed synthetic nanophase akaganeite and lepidocrocite, producing a dark, non-magnetic mineral product that dissolved poorly in oxalate solution. Although the mineral products were spectrally flat and minimally reflective in their VNIR spectra, the FTIR-ATR and Mössbauer spectra and XRD data showed that the minerals present in biotic samples were largely indistinguishable from their parent minerals and the abiotic controls, partially due to the fine-grained nature of the samples, which can mask subtle spectral changes under broadened peak structures. Spectral changes, where identified, could not be matched to any common mineral phase, although various green rusts could not be ruled out. TEM analyses showed the presence of amorphous iron-rich nanospheres (AFNS, 50–200 nm diameter) that were adsorbed to the lath surfaces of the parent akaganeite and lepidocrocite crystals but were absent from the abiotic controls (Figure 9). Interestingly, nanospheres produced in both experiments incorporate solution cations into their structures, but the AFNS in the lepidocrocite experiments are rough-edged, and those in the akaganeite experiments are smoothed. Edge structure has important implications for reactivity (Waychunas and Zhang, 2008). Thus, we anticipate that these two types of nanoparticles will behave and age differently. It is presently not clear if these AFNS are the primary product of the bioreduction of more crystalline minerals by *D. ferrireducens* or if they are intermediate phases to a final end product.

The reduction of more thermodynamically stable Fe(III) (oxyhydr)oxides was less bioreactive than the less thermodynamically stable mineral ferrihydrite in terms of Fe(II) production, but equivalent live cell concentrations were achieved. It is, therefore, interesting that the spectral changes were so subtle. One possible explanation is that the majority of the starting oxide is inaccessible for chemical reduction. Thus, only a thin Fe(III) surface layer is mobilized. This surface-only reaction would lead to two factors affecting spectral transformation: insufficient Fe(II) flux to produce magnetite and minimal changes to the bulk phase that dominate the spectral signature for methods like FTIR and Mössbauer. For these two terminal electron donors, bioreduction manifests as a combination of a drastic color change in the VNIR with an absence of spectral changes in the MIR. One potential explanation for this discrepancy is that the penetration depth of light scales with the wavelength. Thus, VNIR radiation would sample closer to the surface of the grains than MIR radiation. If one assumes a penetration depth of ~10^*^wavelength, then VNIR radiation would sample the top 3.5–25 μm of the sample, and MIR radiation would sample the top 25–250 μm. For reference, the peaks that allow for identifying and discriminating iron (hydro)oxides occur at the longer wavelength end of the MIR spectra range, between 10 and 25 μm, when radiation may be sampling 100–250 μm into the material.

The fact that the AFNS were observed by TEM to attach to the (hydro)oxide surface supports this rationale. However, another factor is the media composition itself. In the pH range of this study, cysteine, which is necessary to instigate bioreduction, is known to immediately absorb the ferrihydrite surface (Cornell et al., 1990) and oxidize to disulfide, reducing the iron oxide. This common minor media component binds to and reduces ferrihydrite more readily than lepidocrocite (Amirbahman et al., 1997). However, the absence of a substantial reduction in the control sample, where cysteine is also present, indicates that reductive dissolution by cysteine is not the controlling difference between these ferrihydrites, lepidocrocite, and akaganeite experimental outcomes.

Geochemical modeling predicted that FeOOH in the presence of 5 mM Fe^2+^ would reduce to magnetite at 90°C but to siderite (FeCO_3_) at 55°C (Supplementary Table S1). When *P. delaneyi* was grown at 90°C on lepidocrocite, the primary mineral product was siderite (Kashyap et al., 2023). When it was grown on akaganeite, the mineral products were magnetite and a vivianite-like (Fe_3_(PO_4_)_2_ · 8H_2_O) phase (Kashyap et al., 2023). XRD studies showed that the mesophilic bacterium *G. sulfurreducens* transformed lepidocrocite and akaganeite into mixtures of magnetite and goethite, with the proportion of magnetite increasing over time (Cutting et al., 2009). The vast difference in products for *D. ferrireducens* grown on these two minerals from the modeling results reveals a need to better understand the biogeochemical interactions in these systems.

## Conclusion

Amorphous Fe-rich nanospheres, previously reported in Antarctic sediments from Blood Falls (Sklute et al., 2022), could be an important natural, moderately stable intermediate in microbially active environments. Further characterization of the compositional range, prevalence, and stability of these AFNS is needed to fully understand the biological cycling of iron and its potential as a morphological indicator of biological involvement (biosignature). Specifically, finding a link between the structural information revealed with TEM and the bulk properties probed with conventional spectroscopic techniques that are easily deployed on other planetary bodies is a crucial step in detecting mineral biosignatures. However, these results also showed that understanding both the geochemical factors affecting mineral transformations and the mechanism of interaction between the analytical technique and the sample is key to correctly interpreting potential biosignatures in laboratory bioreduction experiments.

## Data availability statement

The original contributions presented in the study are included in the article/Supplementary material, further inquiries can be directed to the corresponding author.

## Author contributions

ES: Conceptualization, Data curation, Formal analysis, Funding acquisition, Investigation, Methodology, Resources, Supervision, Validation, Visualization, Writing—original draft, Writing—review and editing. DL: Formal analysis, Investigation, Writing—review and editing. KN: Formal analysis, Investigation, Writing—review and editing. KL: Formal analysis, Investigation, Methodology, Validation, Visualization, Writing—review and editing. MD: Funding acquisition, Investigation, Methodology, Project administration, Resources, Writing—review and editing. JH: Conceptualization, Funding acquisition, Investigation, Methodology, Project administration, Resources, Supervision, Validation, Visualization, Writing—original draft, Writing—review and editing.
