# Supplementary material for: Fe(III) (oxyhydr)oxide reduction by the thermophilic iron-reducing bacterium Desulfovulcanus ferrireducens
Source: Front Microbiol. 2023 Oct 20;14:1272245. doi: 10.3389/fmicb.2023.1272245 (PMC10622975; doi:10.3389/fmicb.2023.1272245)
Supplement: Supplementary file 1 [file Data_Sheet_1.PDF]

Supplementary Material

Fe(III) (oxyhydr)oxide reduction by the thermophilic iron-reducing bacterium *Desulfovulcanus ferrireducens*

Elizabeth C. Sklute[1,2], Deborah A. Leopo[3], Kaylee A. Neat[4], Kenneth J. T. Livi[5], M. Darby Dyar[1,4], James F. Holden[3*]

[1]Planetary Science Institute, Tucson, AZ, United States

[2]Los Alamos National Laboratory, Los Alamos, NM, United States

[3]Department of Microbiology, University of Massachusetts, Amherst, MA, United States

[4]Department of Astronomy, Mount Holyoke College, South Hadley, MA, United States

[5]Department of Materials Science and Engineering, Johns Hopkins University, Baltimore, MD, United States

*** Correspondence:**
James F. Holden
jholden@umass.edu

1 Supplementary Tables and Figures

1.1 Supplementary Tables

Table S1 Predicted equilibration mineral products in modified DSM981 medium at 55°C and 90°C containing $Fe(OH)_3$ (ferrihydrite-like), FeOOH (akaganéite- and lepidocrocite-like), and Fe^{2+}.

Condition	55°C			90°C		
	magnetite	siderite	Ferrite-Mg	magnetite	siderite	Ferrite-Mg
**Ferrihydrite-like (suppress hematite and goethite)**						
100 mmol/l $Fe(OH)_3$ + 0.001 mmol/l Fe^{2+}	1 µmol	0	4.9 mmol	1 µmol	0	7.0 mmol
80 mmol/l $Fe(OH)_3$ + 20 mmol/l Fe^{2+}	20.4 mmol	0	0	21.0 mmol	0	5.7 mmol
**Akaganéite/lepidocrocite-like (suppress hematite)**						
100 mmol/l FeOOH + 0.001 mmol/l Fe^{2+}	0	0	0	0	0	0
95 mmol/l FeOOH + 5 mmol/l Fe^{2+}	0	4.8 mmol	0	4.8 mmol	0	0
	magnetite	siderite	hematite	magnetite	siderite	hematite
**Ferrihydrite-like (no suppression)**						
100 mmol/l $Fe(OH)_3$ + 0.001 mmol/l Fe^{2+}	0	0	51.1 mmol	0	0	52.5 mmol
80 mmol/l $Fe(OH)_3$ + 20 mmol/l Fe^{2+}	0	20.0 mmol	41.0 mmol	0	19.9 mmol	42.1 mmol
**Akaganéite/lepidocrocite-like (no suppression)**						
100 mmol/l FeOOH + 0.001 mmol/l Fe^{2+}	0	0	51.1 mmol	0	0	52.5 mmol
95 mmol/l FeOOH + 5 mmol/l Fe^{2+}	0	4.8 mmol	48.6 mmol	0	4.1 mmol	49.9 mmol

For both temperatures and minerals, less than 0.1 mmol of vivianite was formed when Fe^{2+} was present for all conditions.

Table S2 VNIR absorption positions for original and continuum removed spectra for *D. ferrireducens* grown (H+C) on ferrihydrite (Fh), lepidocrocite (Lep), and akaganéite (Akag), along with heated (HNC) and unheated (NHNC) controls and reference materials. All spectra were analyzed and processed using Bruker's OPUS software with manual continuum removal and automated peak location.

	VIS MAX	FeIII	FeIII	FeIII	FeIII	FeII/FeIII			~1.9		
					Original spectra						
**Ferrihydrite**	0.799			0.535	0.969			1.810	1.938		
Fh H+C	0.740			0.549		1.152					
Fh HNC	0.779		0.494		0.950				1.937	2.198	
Fh NHNC	0.788		0.515		0.915				1.937	2.237	
magnetite						~1.04					
maghemite	0.731		0.475								
hematite	0.745			0.541	0.863		1.428	1.801	1.935		
goethite	0.594<br>0.758	0.419	0.479	0.658	0.950		1.469		1.947		2.397
**Lepidocrocite**	0.622<br>0.793		0.478	0.693	0.953			1.820	1.935	2.195	
Lep H+C	0.551	0.398	0.478								
Lep HNC	0.616<br>0.786	0.414	0.478		0.982				1.932	2.251	
Lep NHNC	0.617<br>0.785	0.414	0.478		0.971				1.935	2.2514	
**Akaganéite**	0.730		0.487		0.915		1.458		1.970		2.456
Akag H+C	0.555	0.420	0.485	0.619							
Akag HNC	0.730				0.908		1.453		1.952		2.452
Akag NHNC	0.730				0.913		1.453		1.957		2.455
green slime	0.548					0.718	1.419		1.943		2.498
vivianite	0.474			0.664					1.956		
siderite	0.707	0.392		0.615		1.044	1.454	1.865	1.984		2.324

Table S2 (cont.) Continuum Removed

	VIS MAX	FeIII	FeIII	FeIII	FeIII	FeII/FeIII			~1.9		
**Ferrihydrite**				0.596		1.126	1.402	1.974	1.932	2.276	
Fh H+C				0.549		1.149		1.810			
Fh HNC				0.543		0.945			1.944		
Fh NHNC				0.560		0.987			1.946		
magnetite			0.536		1.107				1.957	2.241	
maghemite			0.509		1.073						
hematite			0.558	0.676	1.001		1.428		1.934		2.406
goethite			0.515	0.669	1.010		1.438		1.941		2.396
**Lepidocrocite**			0.534	0.694	1.018		1.438	1.803	1.935	2.205	
Lep H+C		0.420		0.751			1.457			2.276	
Lep HNC		0.417	0.525	0.684	0.957		1.513		1.951	2.217	
Lep NHNC		0.416	0.528	0.688	0.973		1.468		1.942	2.209	
**Akaganéite**			0.516	0.656	1.005		1.455		1.966	2.316	2.456
Akag H+C		0.485		0.630					1.968	2.250	
Akag HNC	0.398	0.415		0.653	0.976		1.453		1.947		2.450
Akag NHNC	0.398	0.485		0.659	1.005		1.456		1.953		2.454
green slime		0.427			0.815		1.438		1.934		2.496
vivianite	0.379	0.464			1.002		1.475		1.954	2.377	2.453
siderite		0.486	0.521	0.638	1.069	1.225		1.873	1.985	2.322	2.469

1.2 Supplementary Figures

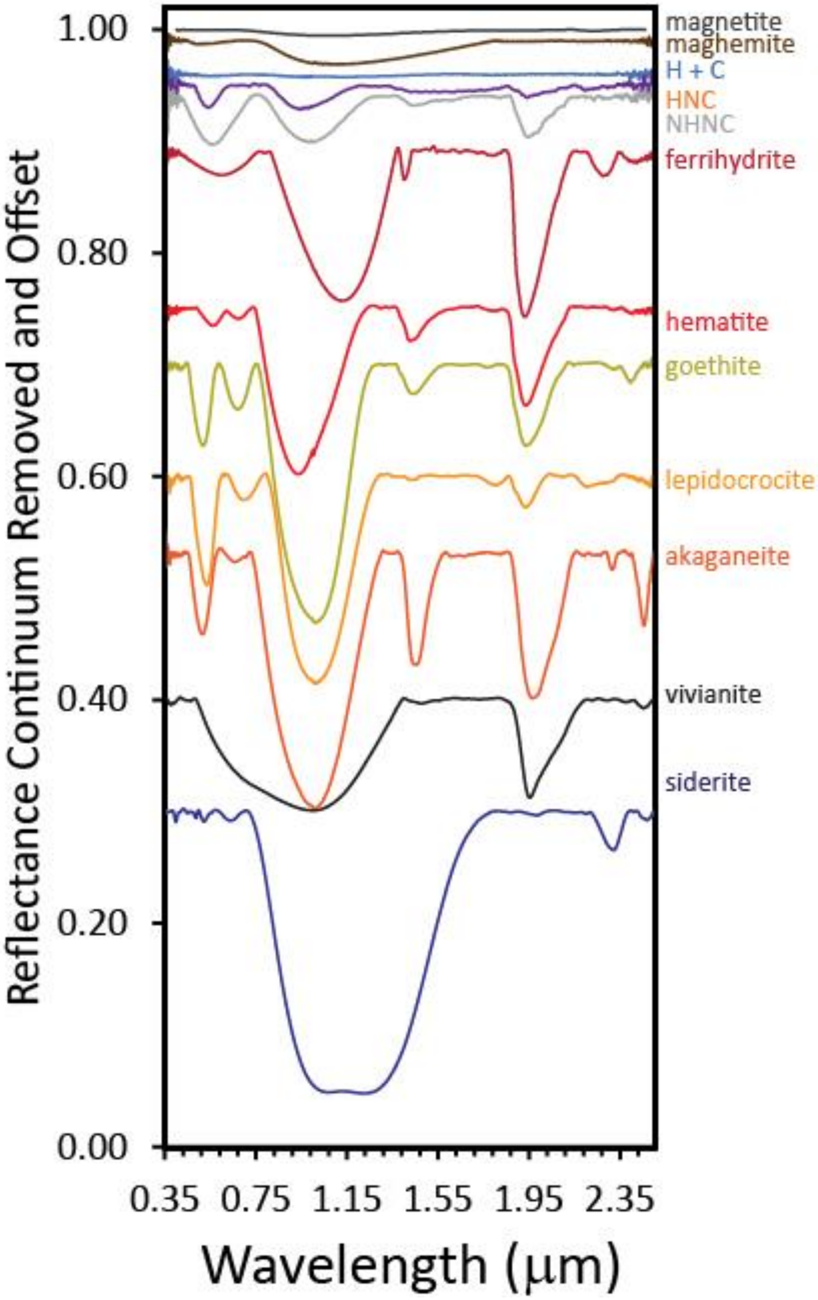

Figure S1 Continuum removed VNIR spectra for *D. ferrireducens* ferrihydrite bioreacted and control samples along with reference materials. The vivianite and siderite spectra are from C-Tape website (https://www.uwinnipeg.ca/c-tape/).

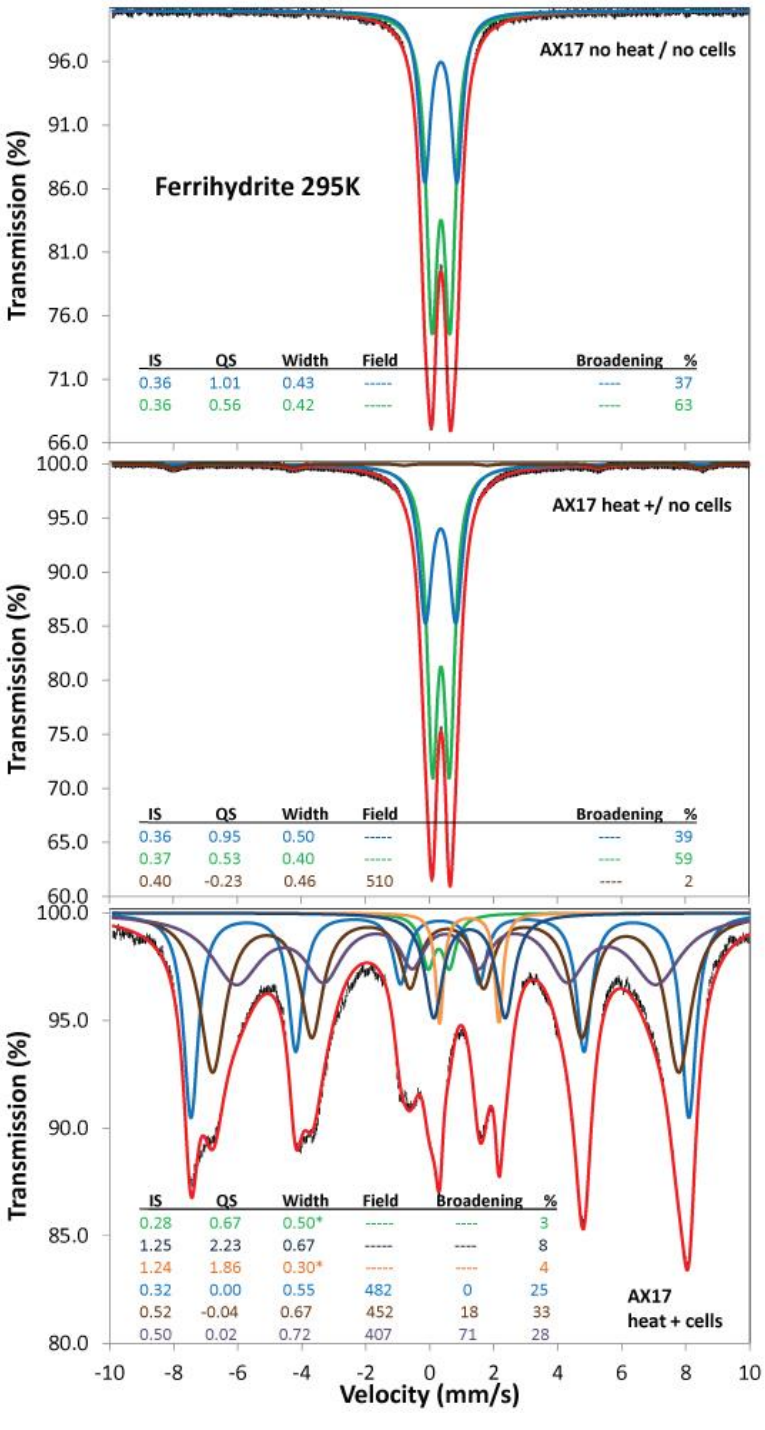

Figure S2 Room temperature Mössbauer spectral fits for *D. ferrireducens* ferrihydrite bioreacted and control samples. Fit parameters for each distribution are shown on the plot with fixed parameters indicated by an asterix. The sum of all distributions is shown in the red line. The experimental data displayed as standard errors for each data point.

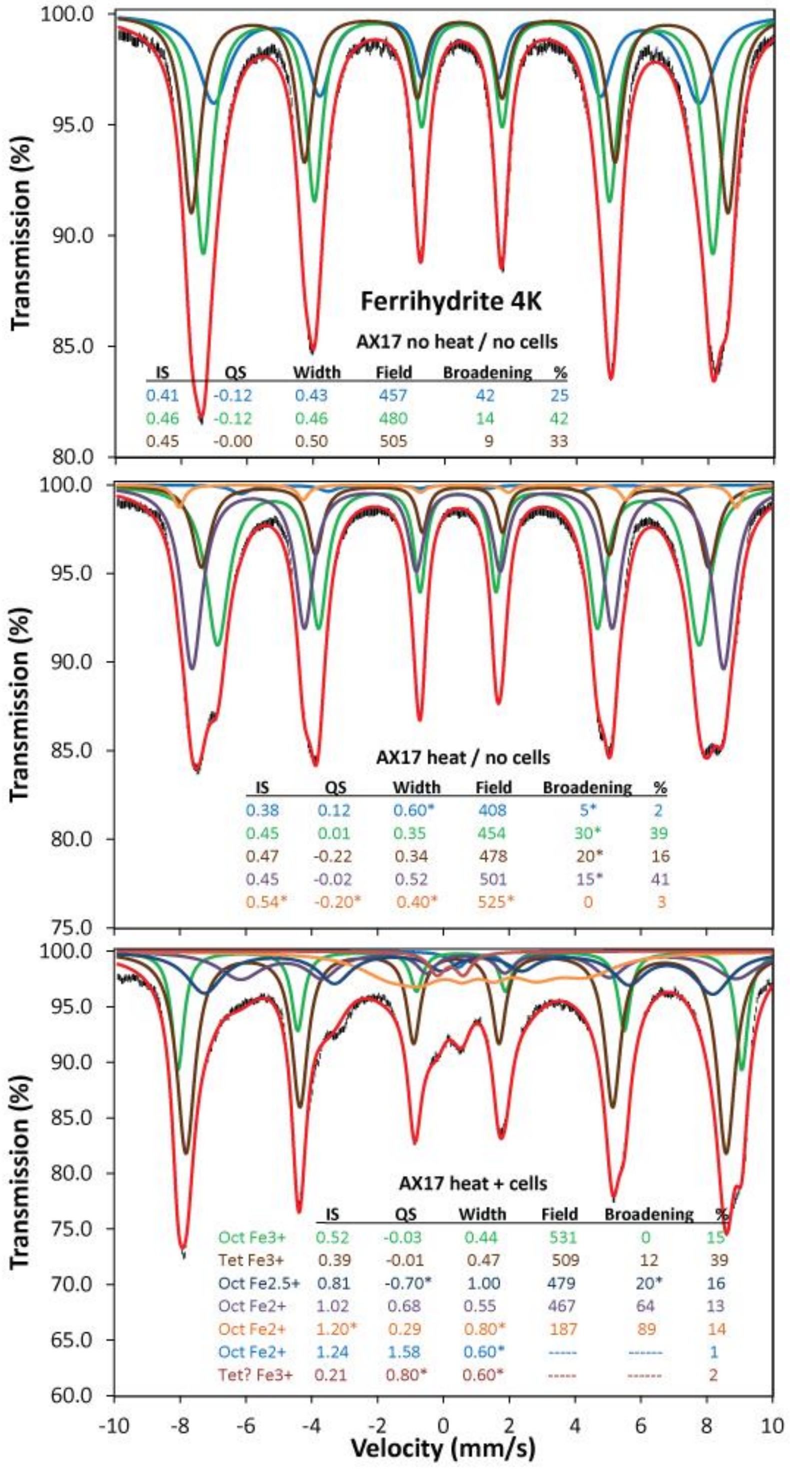

Figure S3 4K Mössbauer spectral fits for *D. ferrireducens* ferrihydrite bioreacted and control samples. Fit parameters for each distribution are shown on the plot with fixed parameters indicated by an asterix. The sum of all distributions is shown in the red line. The experimental data displayed as standard errors for each data point.

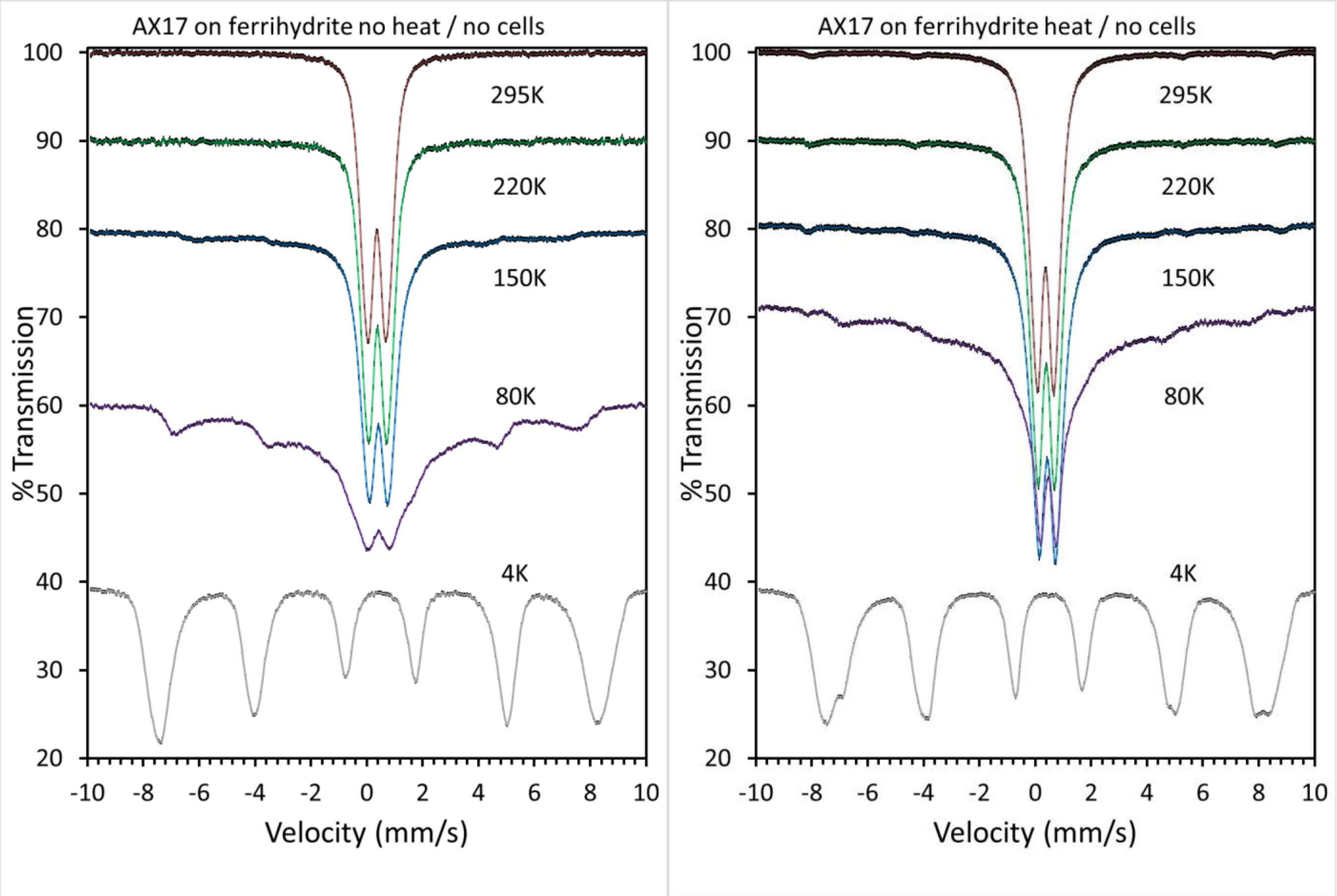

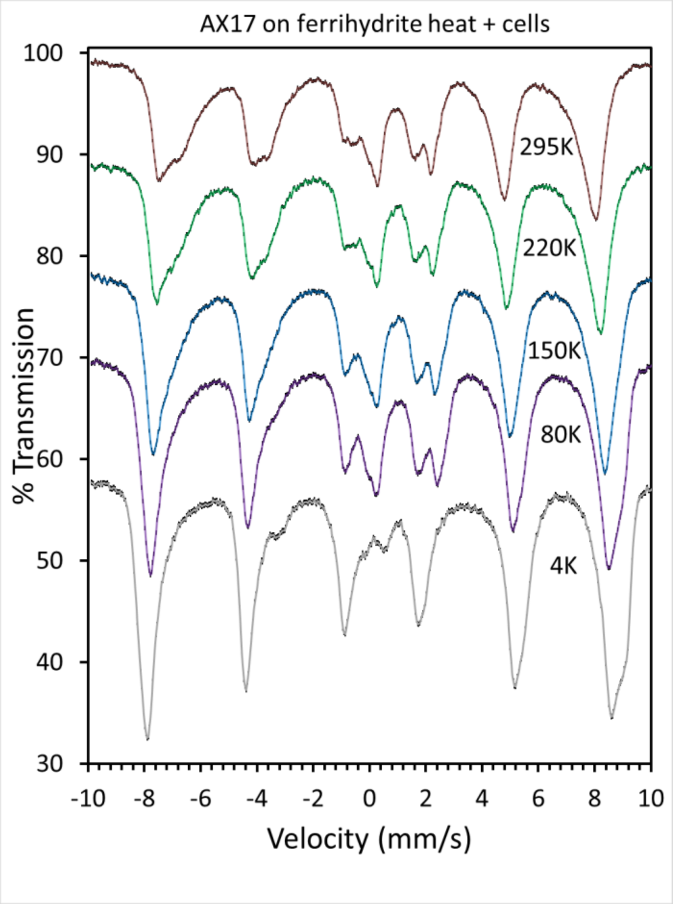

Figure S4 Mössbauer spectra for *D. ferrireducens* ferrihydrite bioreacted and control samples for all temperatures. The magnetic ordering temperature provides additional insight for potential phases. Ferrihydrite and lepidocrocite typically are still doublet at 80K, whereas both control samples are starting to order at this temperature.

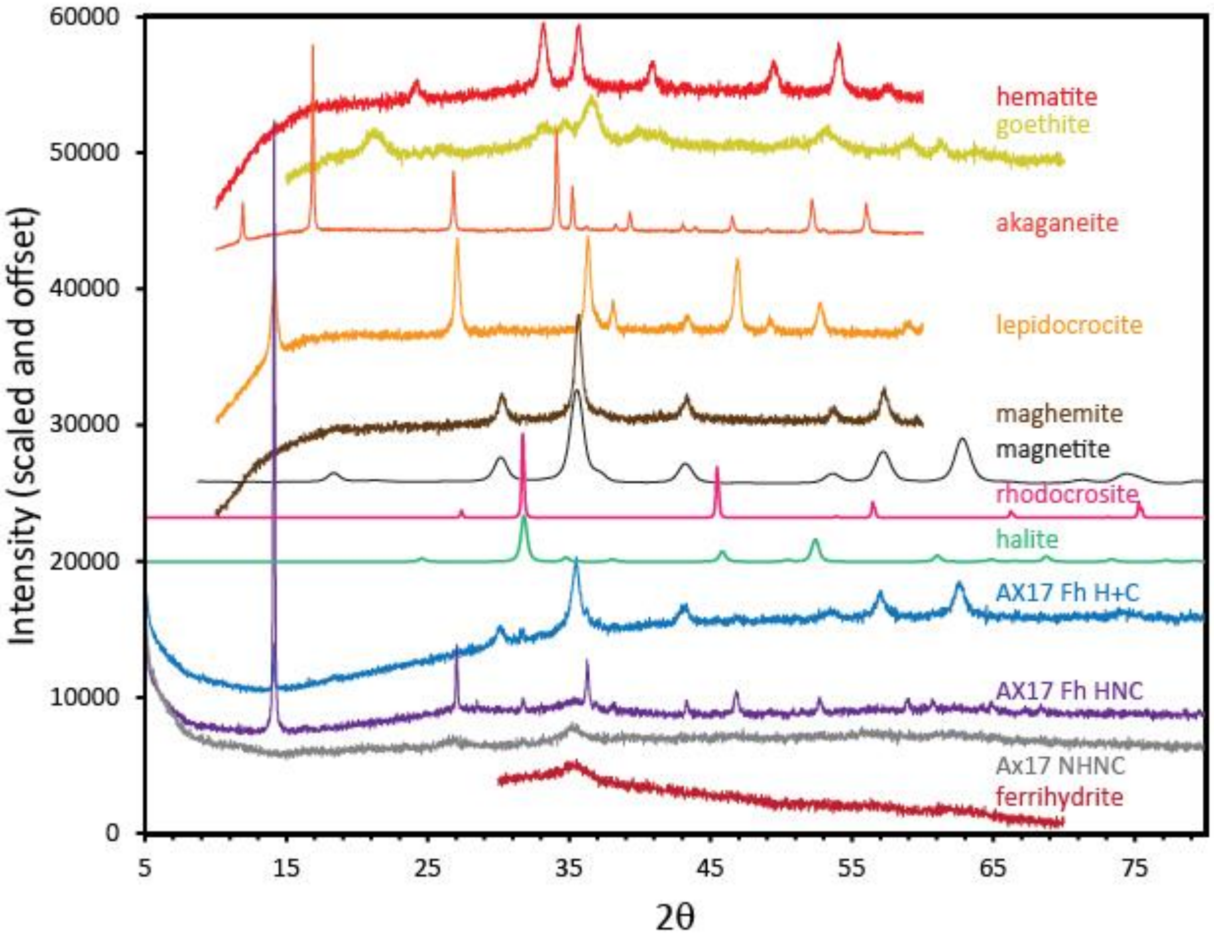

Figure S5 XRD patterns for *D. ferrireducens* ferrihydrite experiments (H+C: heat + cells, HNC: heat/no cells, NHNC: no heat/no cells) along with nanophase iron (oxyhydr)oxides reported in Sklute et al. (2018). Profiles derived for halite 96-900-8679 and rhodochrosite 96-101-1226 from the Match! COD library also shown for reference.

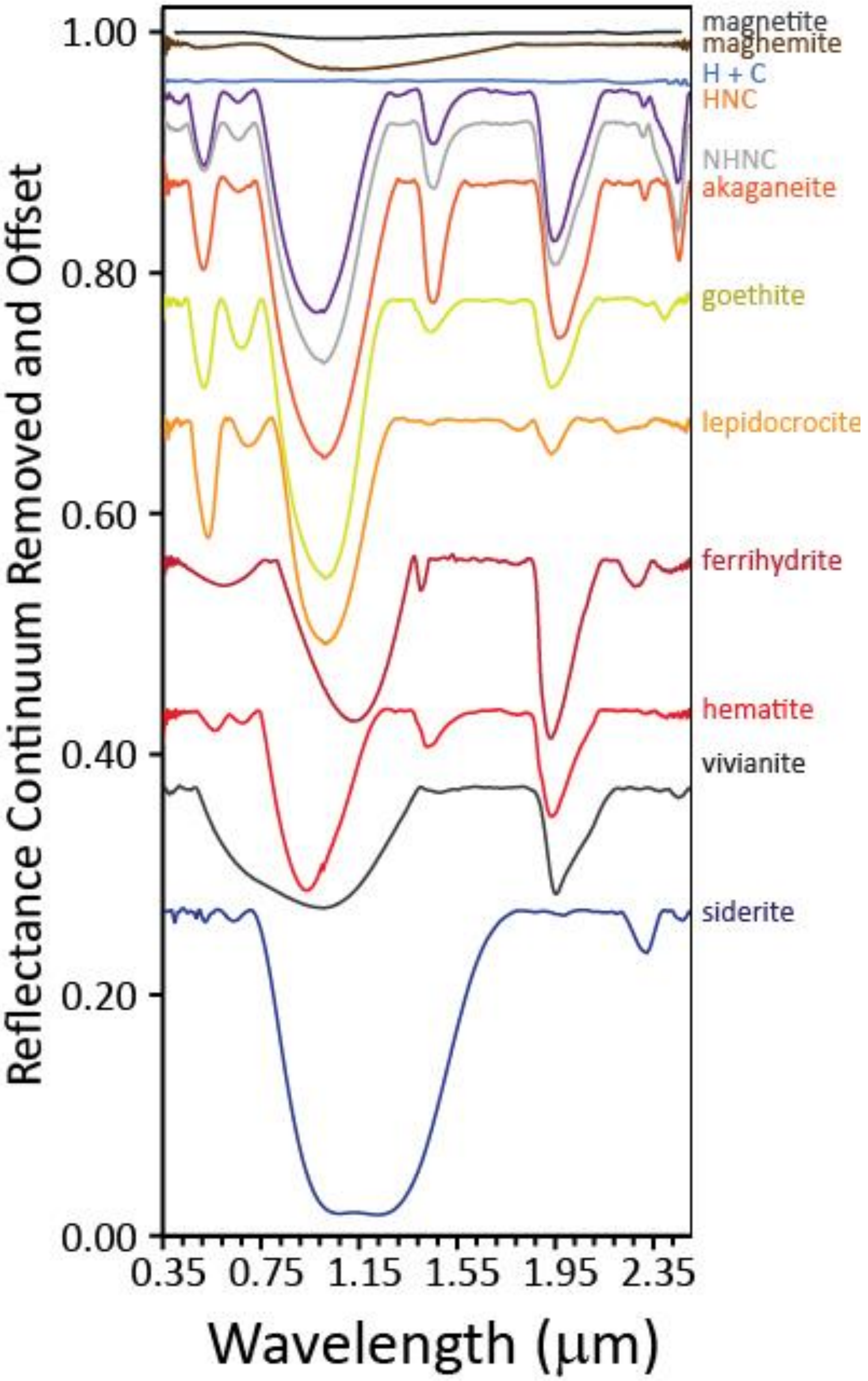

Figure S6 Continuum removed VNIR spectra for *D. ferrireducens* akaganéite bioreacted and control samples along with reference materials. The vivianite and siderite spectra are from C-Tape website (https://www.uwinnipeg.ca/c-tape/). No digital data are available for VNIR of green rust.

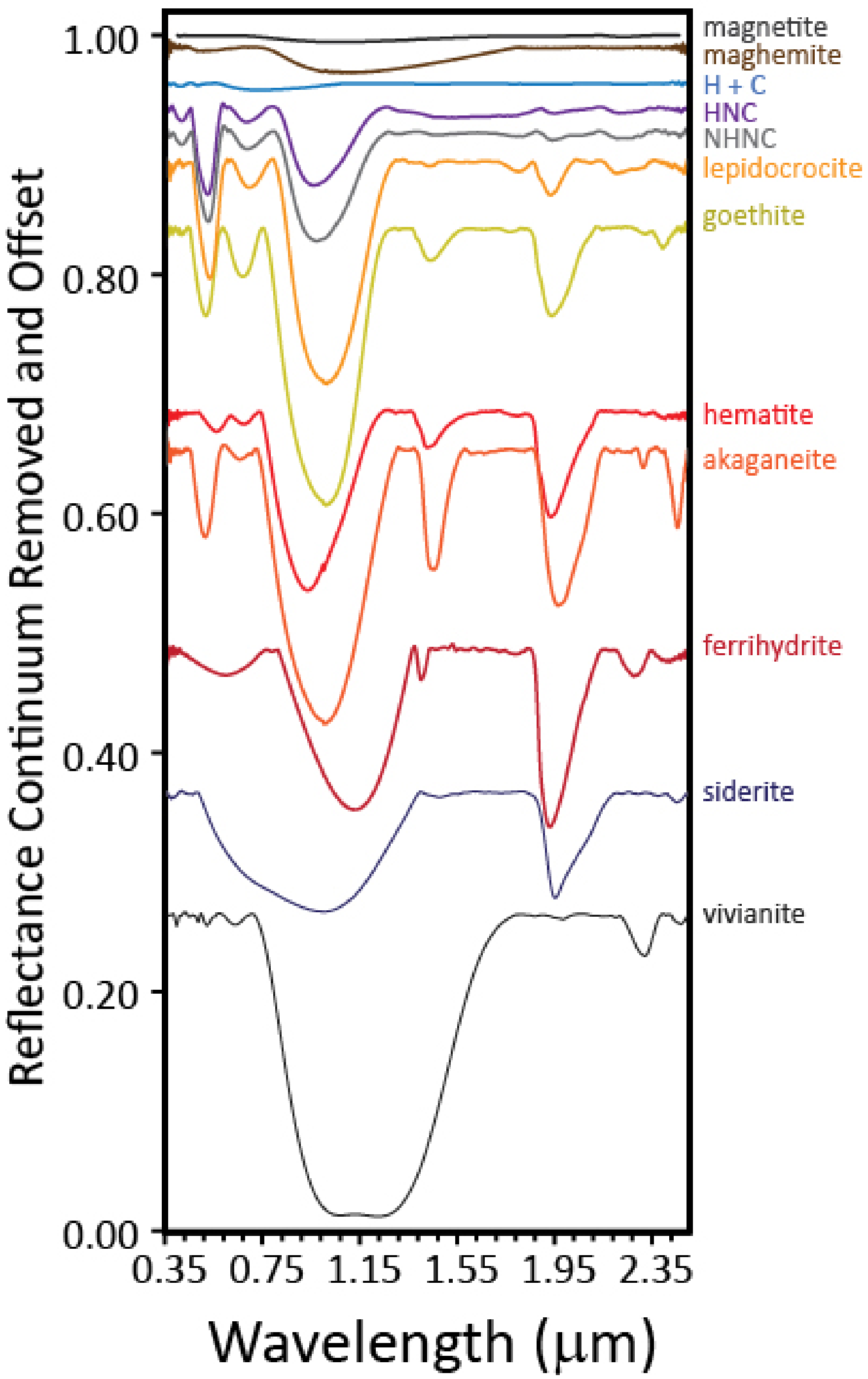

Figure S7 Continuum removed VNIR spectra for *D. ferrireducens* lepidocrocite bioreacted and control samples along with reference materials. The vivianite and siderite spectra are from C-Tape website (https://www.uwinnipeg.ca/c-tape/). No digital data are available for VNIR of green rust.

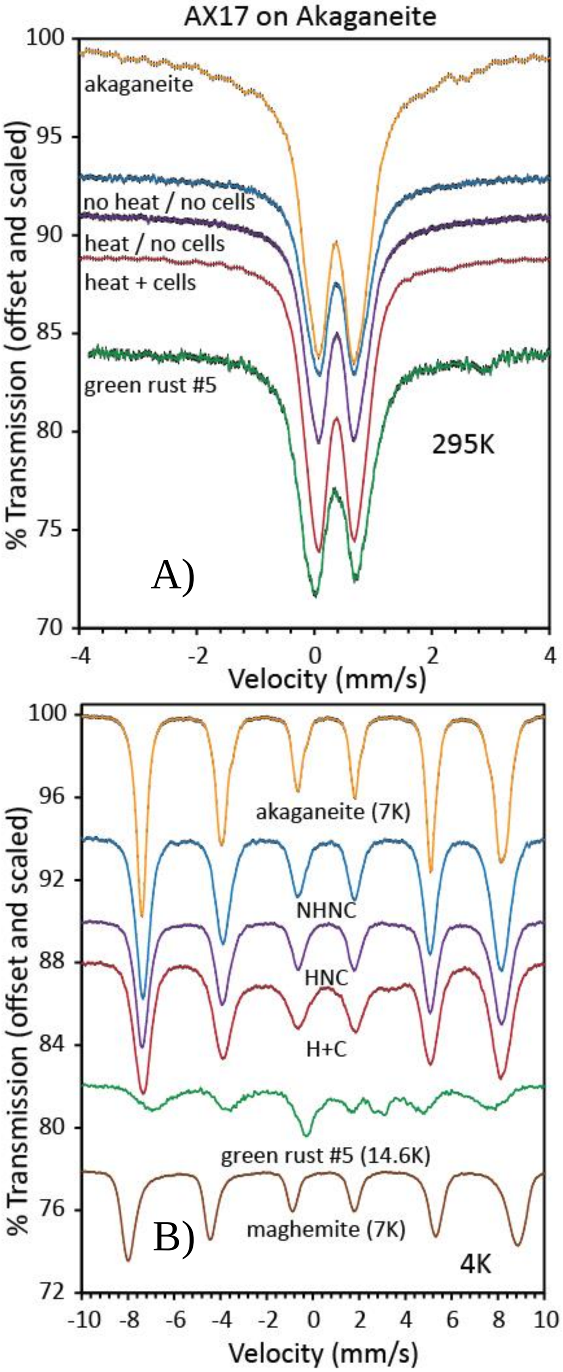

Figure S8 Mössbauer spectra of *D. ferrireducens* grown on akaganéite (heat+cells; H+C), along with heated (heat/no cells; HNC) and unheated (no heat/no cells ; NHNC) controls. (A) 295K spectra for nanophase green rust #5 analyzed for Mazeina et al. (2008; doi:10.1016/j.gca.2007.11.032) and akagenéite along with two control spectra. (B) 4K spectra of *D. ferrireducens* control and bioreacted samples shown with nanophase lepidocrocite (Lep), magnetite (Mag), and ferrihydrite (Fh) taken at temperatures indicated. These are the same Fe(III) (oxyhydr)oxide reference samples studied in Sklute et al. (2018).

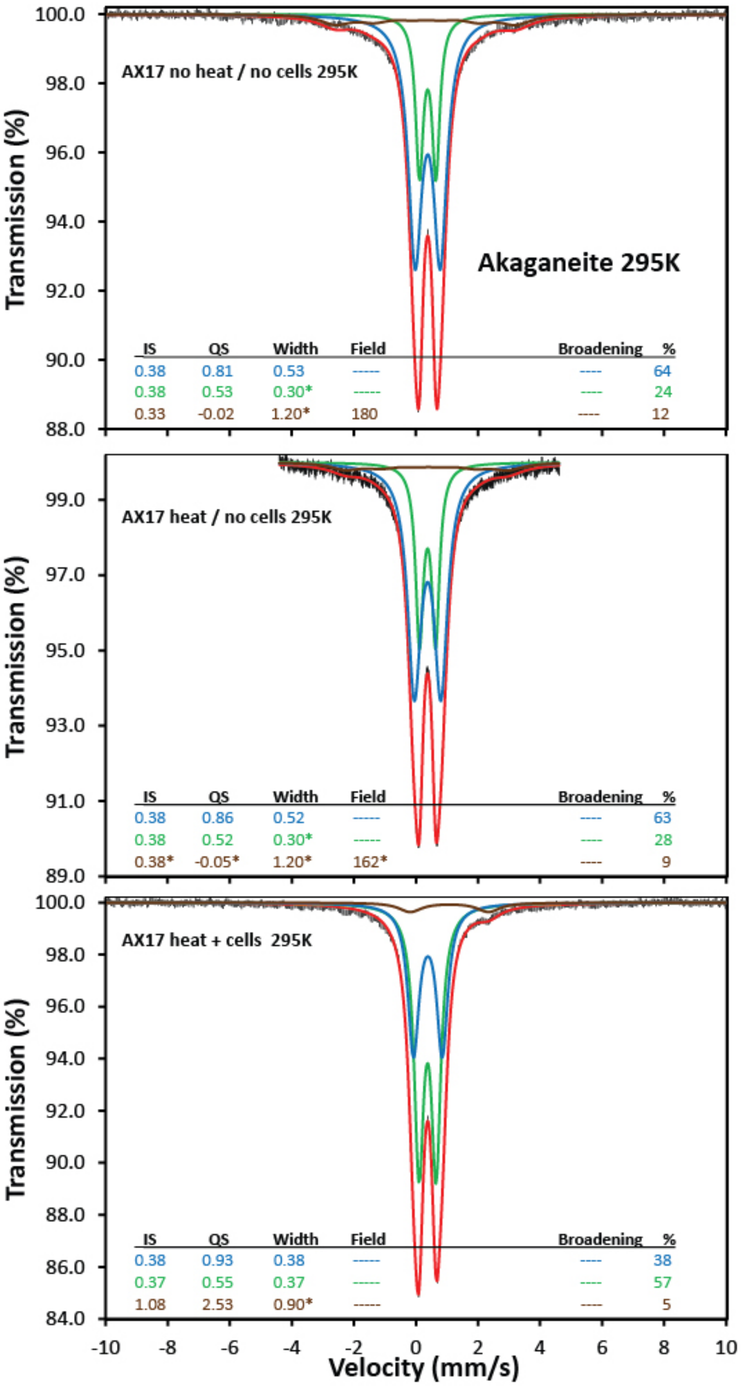

Figure S9 Room temperature Mössbauer spectral fits for *D. ferrireducens* akaganéite bioreacted and control samples. Fit parameters for each distribution are shown on the plot with fixed parameters indicated by an asterix. The sum of all distributions is shown in the red line. The experimental data displayed as standard errors for each data point.

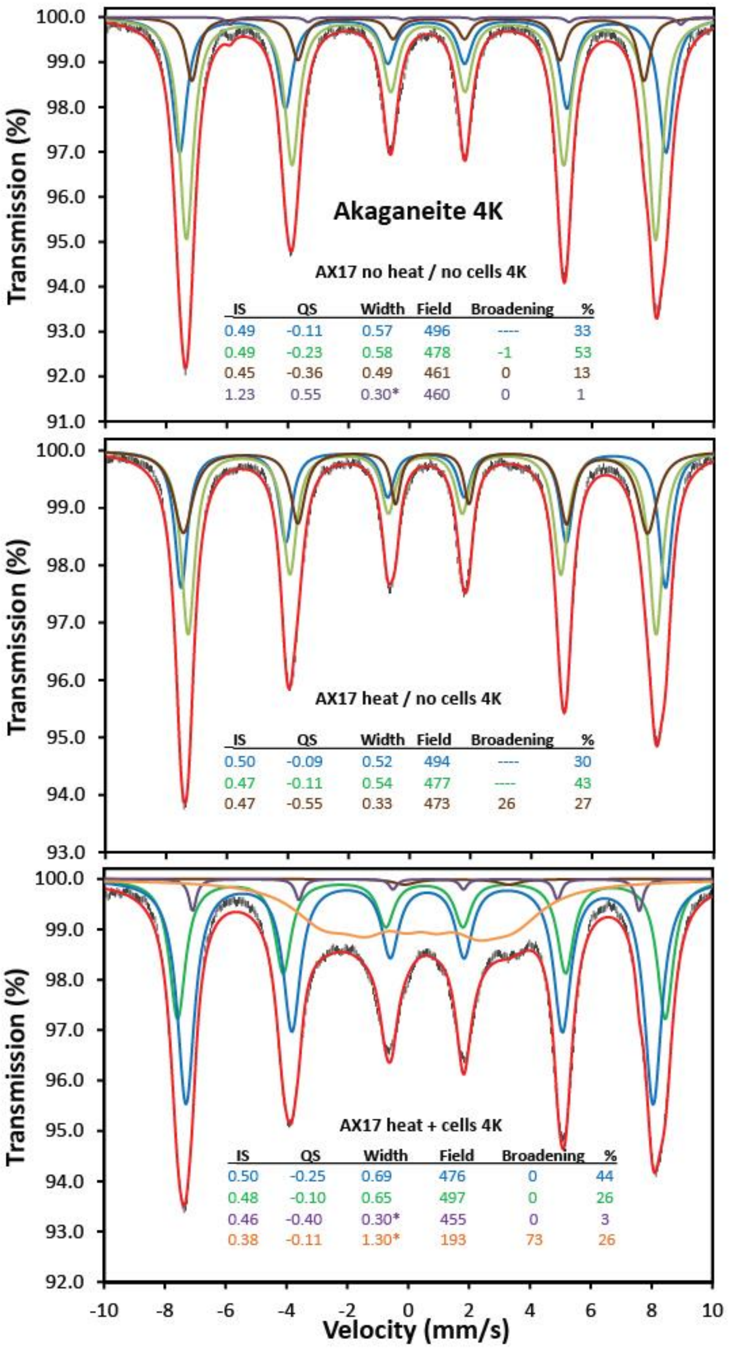

Figure S10 4K temperature Mössbauer spectral fits for *D. ferrireducens* akaganéite bioreacted and control samples. Fit parameters for each distribution are shown on the plot with fixed parameters indicated by an asterix. The sum of all distributions is shown in the red line. The experimental data displayed as standard errors for each data point.

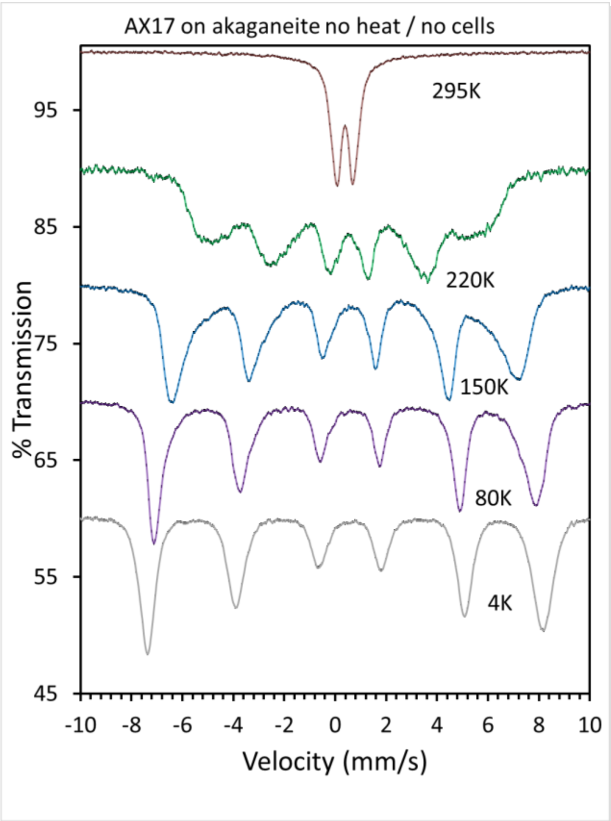

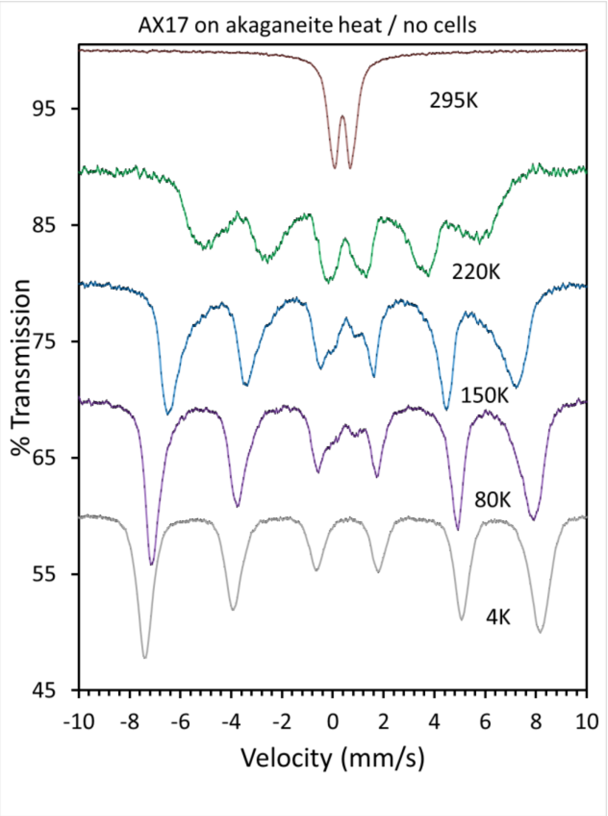

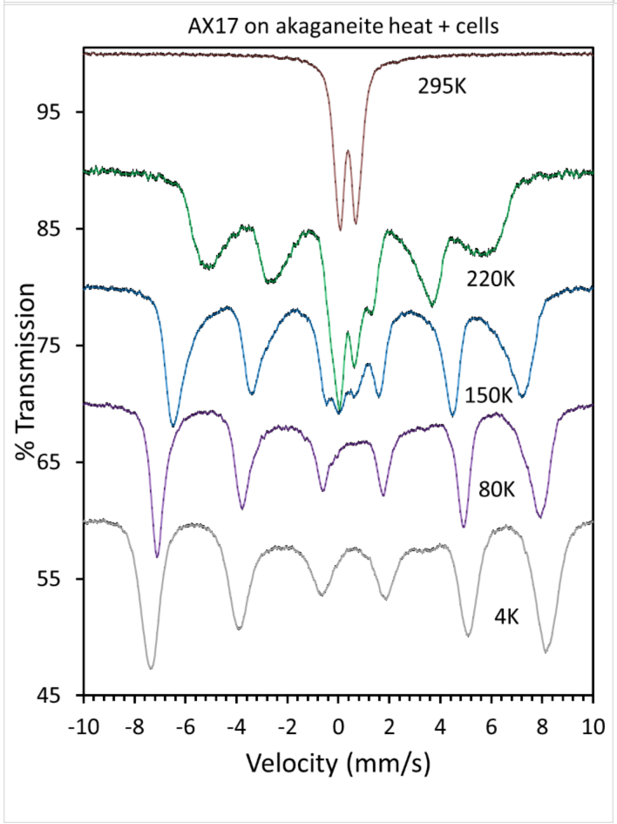

Figure S11 Mössbauer spectra for *D. ferrireducens* akaganéite bioreacted and control samples for all temperatures. The magnetic ordering temperature and provides additional insight for potential phases. Akaganéite starts magnetically ordering by 220K. The persistence of doublets below that point indicates additional phases are formed. Specifically, for the bioreacted sample (bottom left) shows asymmetry in the central doublet that is consistent with admixing of a ferrous phase. This phase cannot be separated adequately from the broad sextets to identify.

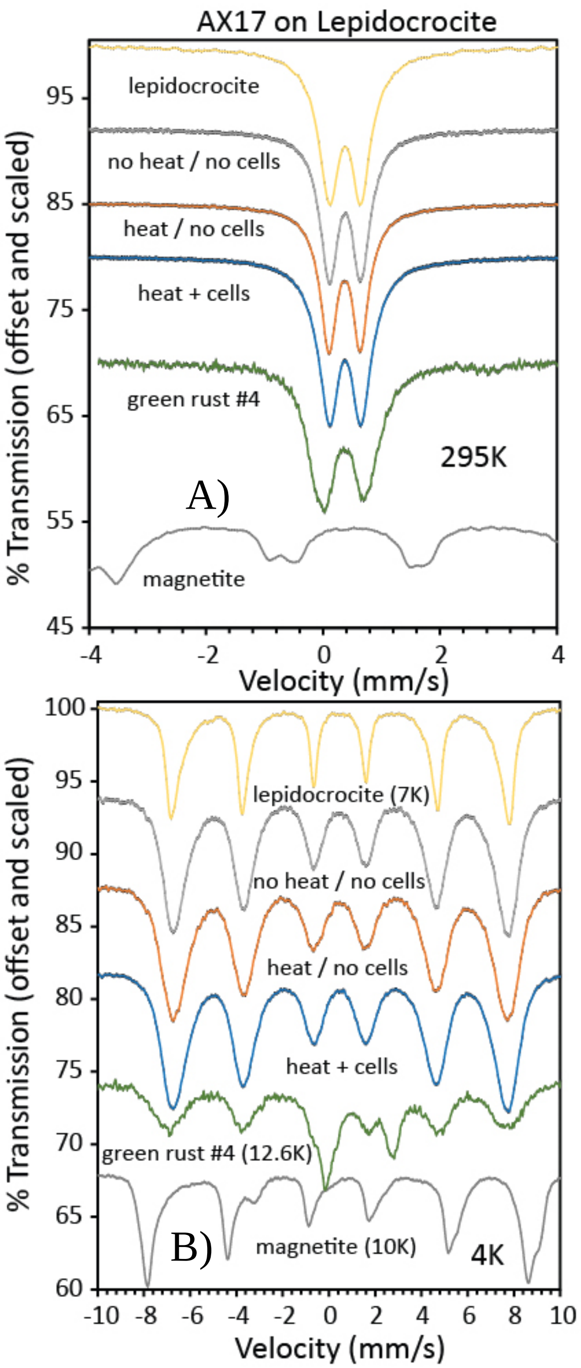

Figure S12 Mössbauer spectra of *D. ferrireducens* grown on lepidocrocite (heat+cells), along with heated (heat/no cells) and unheated (no heat/no cells) controls. (A) 295K spectra for nanophase green rust #4 analyzed for Mazeina et al. (2008; doi:10.1016/j.gca.2007.11.032), nanophase magnetite form Sklute et al. (2018), and lepidocrocite plotted along with two control spectra. (B) 4K spectra of control and bioreacted samples shown with nanophase lepidocrocite, magnetite, and green rust #4 recorded at temperatures indicated on plot.

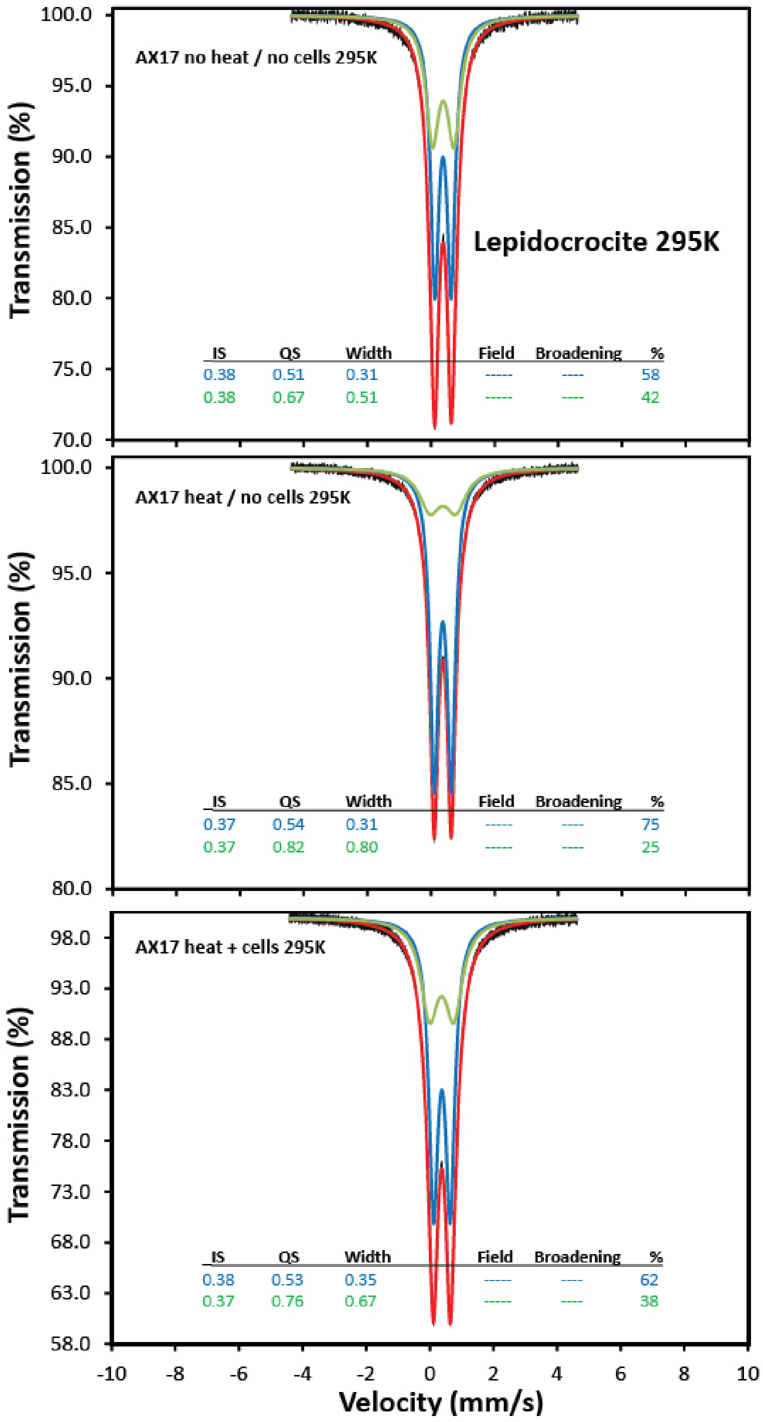

Figure S13 295K temperature Mössbauer spectral fits for *D. ferrireducens* lepidocrocite bioreacted and control samples. Fit parameters for each distribution are shown on the plot with fixed parameters indicated by an asterix. The sum of all distributions is shown in the red line. The experimental data displayed as standard errors for each data point. Relative areas of doublets are likely not diagnostic due to overlap between distributions.

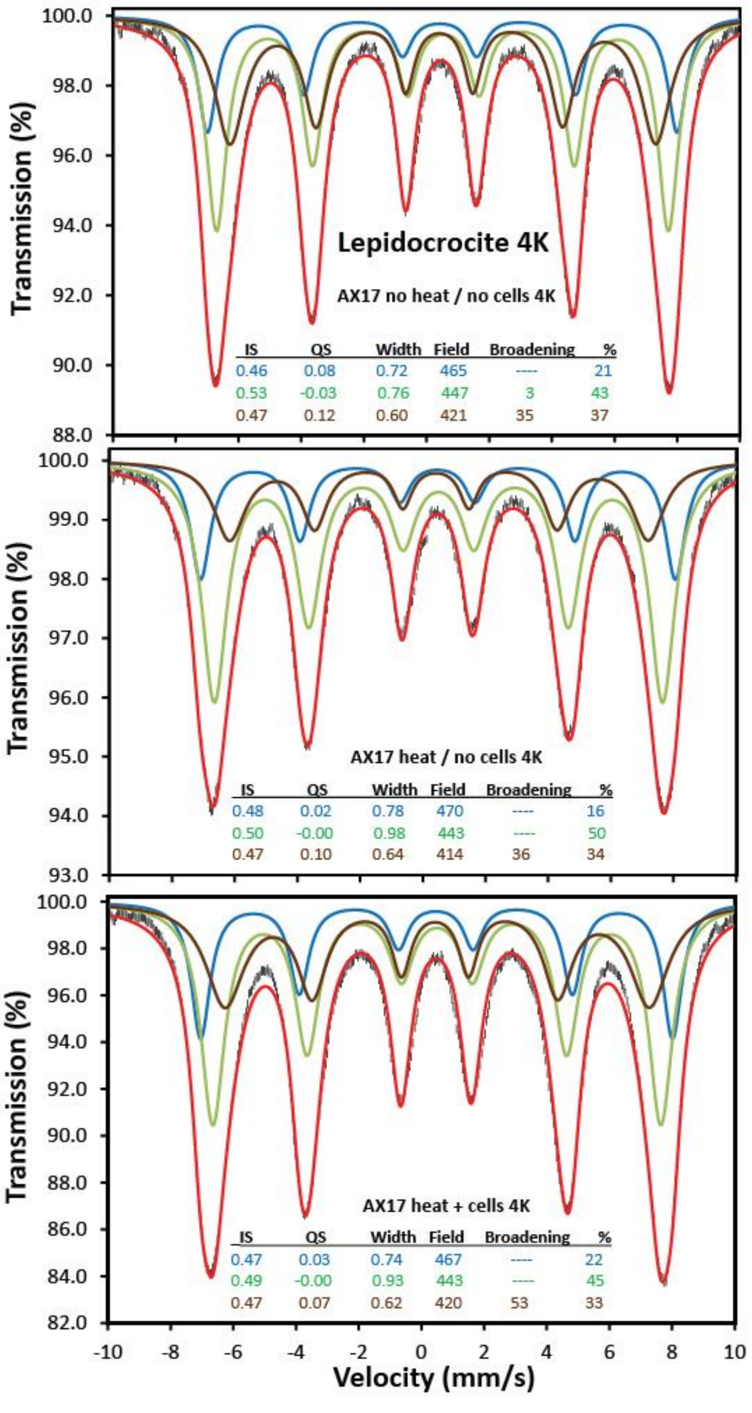

Figure S14 4K temperature Mössbauer spectral fits for *D. ferrireducens* lepidocrocite bioreacted and control samples. Fit parameters for each distribution are shown on the plot with fixed parameters indicated by an asterix. The sum of all distributions is shown in the red line. The experimental data displayed as standard errors for each data point. Only three sextets are used in the heat + cells fit for consistency even though the total fit line does not follow the data between the outer two peaks; this sample likely has a larger distribution of overlapping phases.

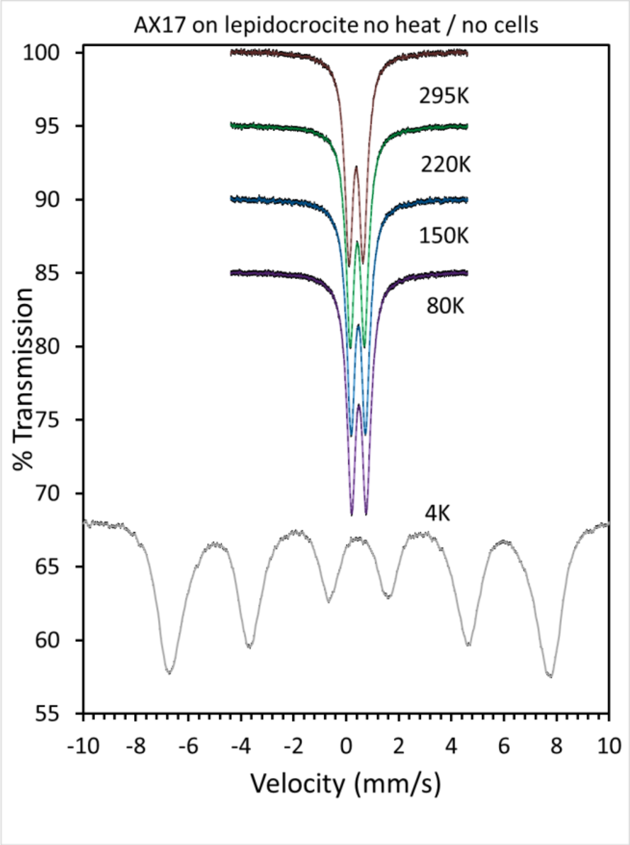

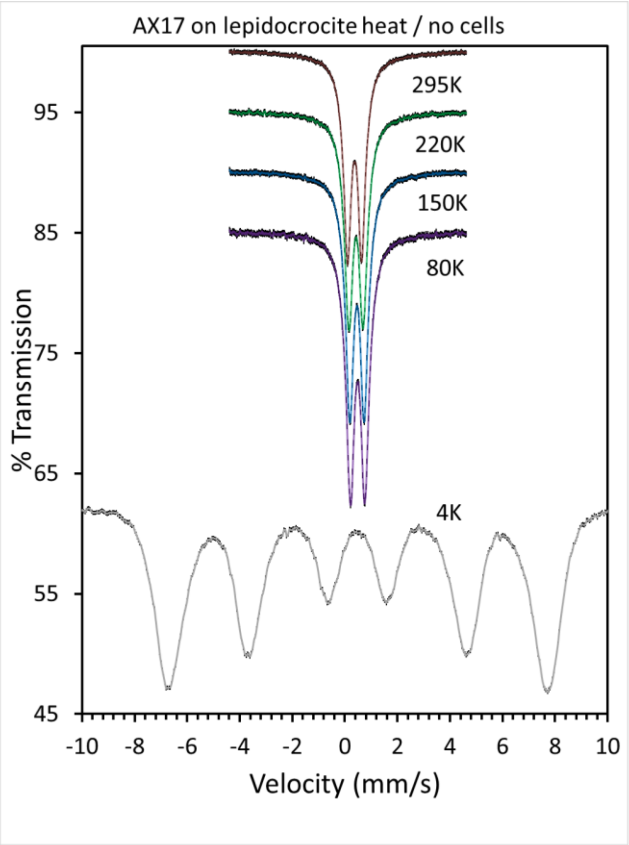

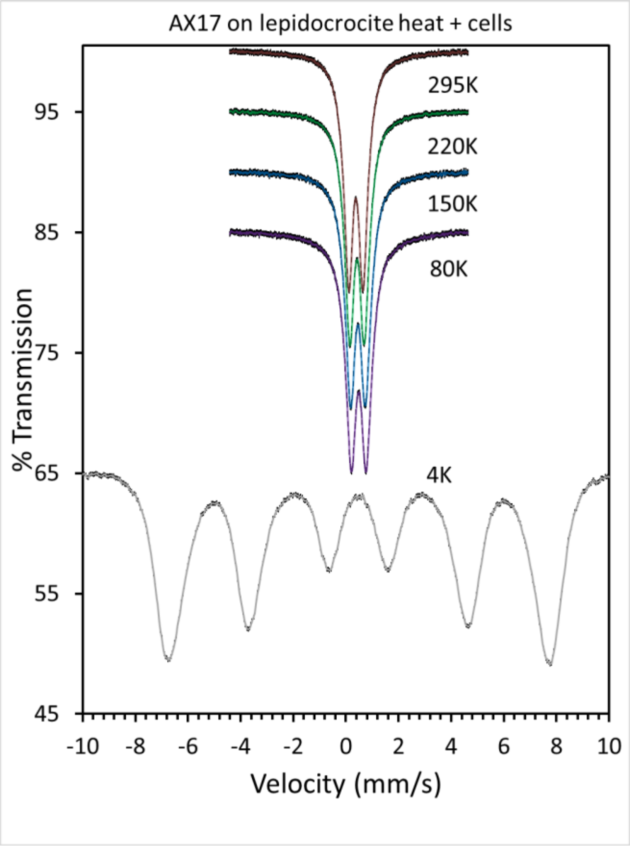

Figure S15 Mössbauer spectra for *D. ferrireducens* lepidocrocite bioreacted and control samples for all temperatures. Despite the distinct color change in the bioreacted sample with respect to the controls, the Mössbauer spectra of all experimental samples at all temperatures appear similar. One possible explanation is that the new phase is a thin coating below the detection limit of the technique. Alternatively, the new phase could be energetically similar to the existing lepidocrocite.

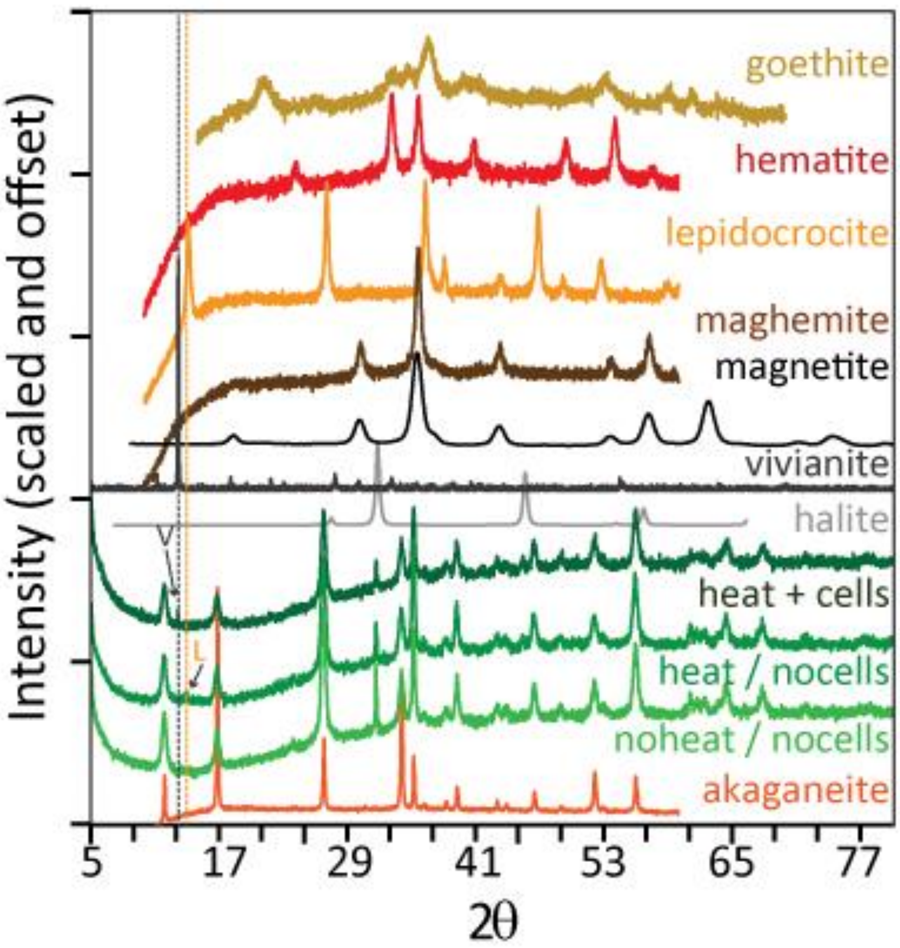

Figure S16 XRD patterns for *D. ferrireducens* lepidocrocite bioreacted and control samples, along with XRD patterns for nanophase oxides from Sklute et al. (2018). The bioreduced and control patterns are nearly identical, and all display a mixture of akaganéite with NaCl. There are two reflections, one in the heated control and one in the bioreduced spectrum that indicate possible additional phases. In the heated control spectrum, there is a reflection ~13.97 2q that is consistent with lepidocrocite. In the bioreduced sample, there is, instead, a reflection ~ 13.16 2q, that is consistent with the main low 2q reflection in vivianite. While one reflection is not enough to identify a phase, trace vivianite could explain some of the color change seen in the bioreduced sample. When vivianite is added as a matching phase, Match! determines it comprises 14.4 mol% of the sample.
